# Elephant-Initiated Interactions with Humans: Individual Differences and Specific Preferences in Captive African Elephants (*Loxodonta africana*)

**DOI:** 10.3389/fvets.2017.00060

**Published:** 2017-04-28

**Authors:** Zoë T. Rossman, Clare Padfield, Debbie Young, Lynette A. Hart

**Affiliations:** ^1^Department of Evolution and Ecology, University of California-Davis, Davis, CA, USA; ^2^African Elephant Research Unit, Knysna Elephant Park, Western Cape, South Africa; ^3^Department of Population Health and Reproduction, School of Veterinary Medicine, University of California-Davis, Davis, CA, USA

**Keywords:** elephants, human–animal interactions, human–animal bonds, social behavior, free contact

## Abstract

South Africa has seen a recent increase in the number of African elephants (*Loxodonta africana*) maintained in reserves and parks and managed in free contact, where they may spend a significant amount of time in close proximity to humans. This study investigates how individual elephants choose to initiate interactions with humans by examining whether interaction types and frequencies vary both between elephants and with regards to the category of human involved in the interaction. Observations were made on a herd of seven captive African elephants frequently exposed to elephant handlers (guides), volunteers (who carry out general observations for the park’s research unit), and tourists. The elephants differed in the frequencies with which they initiated interactions with each category of human and in the types of behaviors they used to initiate interactions. However, all of the elephants interacted most frequently with guides. Certain individual elephants showed preferences in interacting with specific guides, indicating particular elephant-guide bonds. This study provides evidence for elephant-handler bonds as well as information on the extent of interactions between humans and African elephants managed in free contact.

## Introduction

As wild elephant populations decline, many African elephants (*Loxodonta africana*) are maintained in reserves and parks across Southern Africa, where they may spend a significant amount of time in close proximity to humans. There are currently 129 captive elephants in South Africa, which is approximately a 30% increase in the last decade (D. Young, personal communication, 18 February 2016). Captive elephants are maintained in parks and zoos where management techniques range from protected contact to free contact, a method where elephant handlers work alongside elephants with no physical barrier. Despite the large number of elephants living in captive facilities, there is little information on the interactions that take place between captive African elephants and the humans with whom they are in contact.

The effects of human–animal interactions (HAIs) have been studied extensively across a variety of species. Interactions with humans may have the effect of reducing stress in dogs, a quality that interaction with other dogs lacks (1). Dogs also preferred to initiate interactions with humans to kennelmates (2). HAIs have been successful as enrichment for both captive chimpanzees (3) and gorillas (4). Both studies found that increased interaction with humans led to an increase in affiliative behaviors between conspecifics and a decrease in abnormal behaviors or stereotypies. Interactions that are initiated by the animal have further implications. Human-directed behaviors such as approaching or seeking out can be related to how friendly a particular animal is (5). These types of behaviors may be indicators of attachment (6) or even indicators of positive relationships between humans and animals (7).

The human–animal relationship (HAR) and its subset, the human–animal bond (HAB), are two additional concepts that are becoming increasingly important in the field of animal behavior. HABs have been defined as “reciprocal and persistent” relationships that benefit both parties involved (8, 9). The potential for animals to develop HABs has been evidenced in multiple species, including dogs and horses (1), farm animals (10), and various zoo animals (5, 8). A positive relationship has been shown between the frequency of HAIs and the subsequent development of a HAB (1). The implications of the HAB in a captive facility include increased ease of management and potential increase in quality of life for the animals (8).

Personality and temperament play a role in HARs, as the extent to which an animal is willing to interact with humans varies depending on the individual. Personality and temperament are often treated as synonyms and have been defined as the consistent, specific behavior patterns of an individual (11). Elephants specifically have been shown to differ individually in temperament traits relating to social integration, leadership, aggression, and exploratory behaviors (12). The different temperaments of individuals can also be important in determining how animals will interact with humans (6, 13). Individual behavioral variations in response to the presence of a stranger, more specifically exploratory behavior versus fearful behavior, have been demonstrated by house cats (14) and deer (15). Human personality traits may also have an effect on HAIs, as chimpanzees have been shown to differ in their response to humans based on whether the experimenter acted shy or bold (16).

The information on the interactions and relationships between elephants and humans is limited and consists mainly of data concerning Asian elephants. HABs between Asian elephants and their individual handlers, or mahouts, are discussed at length in Ref. (17). Through interviews with mahouts, it was shown that HABs allowed the mahouts to work more safely and productively with their elephants due to the high level of “trust” that had developed over time. Mahouts also specified that their elephants would not necessarily respond to the commands of others, which reinforces the idea that HABs are highly individual. In a separate study, boys as young as 12 were able to work safely with female Asian elephants and even indicated preferences for certain elephants, indicating that the development of HARs with captive elephants is not necessarily limited to trainers who exert control over their animals (18). Following their time spent working with elephants, Lehnhardt and Galloway (19) described the ability of HABs between trainers and elephants to contribute to the safety of training and handling elephants and indicated that the formation of the HAB may be more important than any formal training on how to handle elephants. This study also acknowledges the lack of information on HAIs between humans and young, male African elephants.

Elephants are cognitively advanced creatures with the largest brain of any land mammal and a remarkable capacity for long-term social memory (20). From their reactions to injured conspecifics (21) to their ability to recognize the calls of an estimated 100 other individuals (22), it is clear that interactions and relationships with conspecifics play an important role in the day-to-day life of the African elephant. Positive interactions occur frequently between females and calves in a herd (23) and families often function cohesively (12).

In a captive setting, African elephants have been shown to vary in their individual personalities, and various methods of rating temperament traits in elephants have proven successful (24, 25). These studies also discuss how recognizing an individual elephant’s unique set of characteristics can help direct management practices that cater to an elephant’s particular needs. For Asian elephants, mahouts similarly identified specific traits that they found either preferable or undesirable concerning the handling of a working elephant (17). The variability in an individual elephant’s personality may then inform the frequency and types of interactions that they exhibit toward humans, as previously discussed regarding other species.

The purpose of this study is to provide an in-depth look at how individual captive African elephants in a free contact environment choose to initiate interactions with humans, and whether interaction types and frequencies vary both between elephants and with regards to the type of human involved in the interaction. At the study site, it is anecdotally accepted that certain elephants maintain unique bonds with certain handlers, and that some elephants are friendlier overall than others. Past observations at this site provided further indications that the focal elephants may differ in when and how they interact with humans, and with which humans they choose to interact. This study attempts to use detailed information collected on elephant-initiated interactions in order to address the following:

- Will elephants show variations both individually and as a group in the frequencies and types of interactions they initiate overall?
- Will elephants show variations both individually and as a group in the interactions initiated toward a specific category of human (handler, volunteer, or tourist)?
- Within the subset of elephant-handler interactions, will elephants show individual variations in the numbers of interactions initiated toward particular handlers, indicating potential human–elephant bonds?

## Materials and Methods

Observations were conducted on a herd of seven captive African elephants (*Loxodonta africana*) at Knysna Elephant Park (KEP), Western Cape, South Africa. KEP was home to 18 elephants when the study was conducted; however, only seven of them make up the herd that interacts with tourists, hereafter referred to as Sally’s herd (see Table 1 for a full herd profile). Sally’s herd is composed of five females and two males ranging in age from 7 to 25 years old. The elephants come from a variety of backgrounds—two of the elephants, Shungu and Thandi, were born at KEP, and the other five are rescued orphans. Only two of the elephants are related: Nandi and daughter Thandi. Despite their individual past circumstances, the members of Sally’s herd still appear to function in a relatively similar fashion to herds in the wild, with a matriarch and clear hierarchical order among the elephants that affects group decision-making. The elephants in the herd maintain strong intraspecific relationships; therefore, they have no evident need to fill any void left by lack of social contact with other elephants. However, even the most social elephants in the herd choose to interact with humans, making these human–elephant interactions especially interesting.

**Table 1:** **Sally’s herd profile**.

Name	Position in hierarchy^b^	Sex	Age	Additional information
Sally^a^	1	F	25	Matriarch
Nandi^a^	2	F	22	Mother of Thandi
Thandi^a^	3	F	11	Daughter of Nandi, born at Knysna Elephant Park (KEP)
Keisha	4	F	11	
Mashudu	5	M	7–8	
Thato^a^	6	F	7	
Shungu	7	M	8	Born at KEP

*^a^Members of the core social group within the herd*.

*^b^Average values for position in hierarchy were determined through discussion with staff from AERU and KEP*.

The herd is managed in free contact, and during park hours, the elephants are regularly exposed to elephant handlers (hereafter referred to as guides), tourists, and volunteers. Tourists generally arrive up to once every half hour, when they may bring buckets of fruit and vegetables to feed to the elephants from across a barrier. After this, the tourists (accompanied by guides) are allowed to walk with, touch, and take photos with the elephants. Volunteers collect data for KEP’s African Elephant Research Unit (AERU) and spend around 4 h per weekday in close proximity to the elephants. Their direct contact is more limited as their time in the field is spent collecting general behavioral data on the elephants. Volunteers are required to commit to a minimum of 2 weeks at the park; however, some stay up to 3 months. The guides spend a great deal more time in close proximity to and in contact with the elephants than either volunteers or tourists do. In addition to the time spent moderating tourist–elephant interactions in the field, the guides spend extra time with the elephants, training and riding them. The length of time that the guides have been at the park ranges from several months to 20 years.

Observations were made on interactions between the seven elephants and three types of humans by a single experimenter (Zoë T. Rossman). Interactions were defined as behaviors that were clearly directed toward a specific human. Only interactions that were initiated by the elephant and not directly motivated by food were recorded. Elephant-initiated interactions were defined as interactions that an elephant chose to initiate and interactions that did not follow a command from a guide. Direct food-motivated interactions were defined as any interactions taking place during periods where the elephants were behind the feeding barrier, and any interactions with guides carrying pellet bags, as these were the two situations in which the elephants expected a food reward for an interaction. These interactions were ignored since the elephant’s motivation for interacting was directly and inextricably linked to the promise of a food reward, and thus of little relevance to this study.

Data on interactions were collected from June to September 2015 over 243 h of direct observation. Observations were made daily in intervals between 9:00 a.m. and 4:00 p.m. A roughly even number of observations were conducted during the first half of the day and second half of the day to control for any potential daily variations in the herd’s routine. An all occurrences sampling method was used (26), and Zoë T. Rossman moved position as needed in order to keep the maximum number of elephants in view. Final interaction counts were adjusted for how long each elephant was visible in the focal group (i.e., the members of Sally’s herd within viewing distance). However, differences in time in view for each elephant were negligible (<5%). For every interaction, the time, identity of the elephant, and behavior were recorded, as well as the type of human (guide, volunteer, tourist) and coded identity of the human (if guide). Number of tourists in the field was also recorded, as well as which elephants were visible in the focal group.

Behaviors used to initiate interactions were mainly sourced from a working AERU ethogram and modified in order to account for the human target of the behavior. Thirty individual behaviors were used by the elephants to initiate interactions with humans; these behaviors were grouped into six categories: “trunk out,” “trunk to human,” “trunk to object on human,” “seeking out,” “prolonged contact,” and “other.” Categories were based on the similarities of individual behaviors. “Trunk out,” “trunk to human,” and “trunk to object on human” behaviors are all short, exploratory behaviors, whereas “seeking out” and “prolonged contact” behaviors indicate a higher level of commitment from the elephant to the interaction. The “other” category is made up of rare behaviors where the intention of the elephant is unclear: for example, potentially agonistic or playful behaviors. A full list of behaviors and descriptions is available in Table 2.

**Table 2:** **Behaviors**.

Group	Behavior	Description
Trunk out	Trunk out	Trunk extended outwards and held for at least 2 s toward human
Trunk to human	Trunk to body	Trunk tip touches human’s torso or back
	Trunk to arm	Trunk tip touches human’s arm (above the wrist)
	Trunk to hand	Trunk tip touches human’s hand (below the wrist)
	Trunk to leg	Trunk tip touches human’s leg (above the ankle)
	Trunk to foot	Trunk tip touches human’s foot (below the ankle)
	Trunk to head	Trunk tip touches human’s face or head
	Other touch	Trunk touches human in a way that does not fall under other “trunk to human” behaviors
Trunk to object on human	Trunk to personal item	Trunk tip touches human’s personal item (e.g., purse, camera)
	Trunk to bull hook	Trunk tip touches guide’s bull hook
	Trunk to cane	Trunk tip touches guide’s cane
Seeking out	Approach	Walk toward human, approaching within 2 m (subsequent approaches that occur within 2 min of an initial approach are recorded as “follows”)
	Follow	Start walking or change direction to follow human who walks away at least 5 paces
Prolonged contact	Head lean	Gently lean head against human for at least 2 s
	Hug	Wrap trunk around human’s waist or over human’s shoulder
	Trunk to hand prolonged	Trunk tip or more touches human’s hand (below the wrist) for at least 10 s
	Trunk to arm prolonged	Trunk tip or more touches human’s arm (above the wrist) for at least 10 s
	Trunk to body prolonged	Trunk tip or more touches human’s torso or back for at least 10 s
Other behaviors	Mock charge	Approach human quickly, with ears out
	Push	Displace human with body or trunk
	Trunk flick	Flick trunk forcefully toward human without making contact
	Trunk hit	Deliberately strike human with trunk
	Tail hit	Deliberately strike human with fast slap of tail
	Kick	Deliberately kick human with foot
	Branch throw	Throw branch in direction of human
	Head-to	Turn head toward human, without movement of hind legs
	Face	Turn whole body and head toward human, taking at least 3 steps with hind legs
	Turn body	Turn whole body, taking at least 3 steps with hind legs, but does not turn head
	Ears out	Both ears pulled forward and held there for at least 5 s, directed toward human in front of elephant (not held open by wind)
	Ear flap	Both ears moved forward and back, directed at human in front of the elephant (not associated with cooling)

Data were analyzed using canonical correspondence analysis (CCA) (27) and chi-squared tests for tests of independence between particular categorical variables. These tests were performed using the statistical software SAS 9.4. Total interaction numbers were adjusted to account for the amount of time each elephant was observable. Interactions that were initiated toward a group of people were not included in the analysis as it was difficult to accurately determine who, if anyone, was the actual target of the behavior. In all of these analyses, the individual elephants and guides were treated as fixed effects, since the small sample sizes realistically preclude drawing conclusions about any larger population of elephants or humans.

## Results

### Variation in Types and Frequencies of Interactions

**Figure 1:**
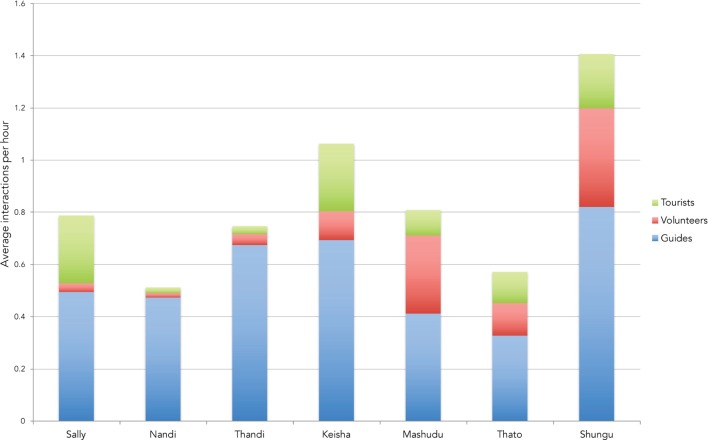
**Average interactions per hour initiated toward each type of human**. The figure shows the average number of interactions per hour initiated by each elephant toward three specific categories of humans: tourists, volunteers, and guides. This graph adjusts for the amount of time each individual elephant was visible in the focal group. Individual elephants differed significantly in the overall number of behaviors exhibited toward any human (*p* < 0.0001). Guide total was significantly greater than volunteer or tourist total (*p* < 0.0001 in both cases), volunteer and tourist totals did not differ significantly (*p* = 0.997).

Figure 1 illustrates the breakdown of the total number of interactions per hour initiated by each elephant on average. The herd exhibited certain groups of behaviors more frequently than others (*p* < 0.0001, see Table 3). Individual elephants differed significantly in the overall number of behaviors exhibited toward any human (*p* < 0.0001, see Table 4). Among the elephants, Shungu interacted the most, initiating an average of 1.4 interactions per hour, and Nandi initiated the fewest interactions, at an average of 0.51 per hour.

**Table 3:** **Behavior group vs. type of human**.

	Guides	Volunteers	Tourists	Total^c^
Trunk out	295 **(33^a^)**	52 **(22)**	85 **(37)**	432 **(32)**
Trunk to human	314 **(35)**	131 **(56)**	116 **(50)**	561 **(41)**
Trunk to object	54 **(6)**	15 **(6)**	15 **(7)**	84 **(6)**
Seeking	182 **(20)**	26 **(11)**	5 **(2)**	213 **(16)**
Prolonged contact	18 **(2)**	4 **(2)**	4 **(2)**	26 **(2)**
Other	43 **(5)**	4 **(2)**	5 **(2)**	52 **(4)**
Total^b^	906	232	230	1,368

*^a^Numbers in bolded parentheses are percentages of each behavior group out of total interactions exhibited toward a specific type of human, not always 100% due to rounding*.

*^b^Guides total was significantly greater than volunteer or tourist total (*p* < 0.0001 in both cases), volunteer and tourist totals did not differ significantly (*p* = 0.997)*.

*^c^Totals differ significantly across all behaviors (*p* < 0.0001) or across four most common behaviors (trunk out through seeking, *p* < 0.0001)*.

**Table 4:** **Behavior group vs. elephant**.

	Sally	Nandi	Thandi	Keisha	Mashudu	Thato	Shungu	Total^c^
Trunk out	49 **(27^a^)**	47 **(39)**	51 **(29)**	87 **(35)**	61 **(32)**	31 **(23)**	106 **(33)**	432 **(32)**
Trunk to human	106 **(58)**	38 **(31)**	60 **(34)**	106 **(43)**	82 **(43)**	55 **(41)**	114 **(36)**	561 **(41)**
Trunk to object	17 **(9)**	12 **(10)**	15 **(9)**	8 **(3)**	9 **(5)**	10 **(8)**	13 **(4)**	84 **(6)**
Seeking	3 **(2)**	18 **(15)**	35 **(20)**	28 **(11)**	33 **(17)**	22 **(17)**	74 **(23)**	213 **(16)**
Prolonged contact	0 **(0)**	0 **(0)**	5 **(3)**	5 **(2)**	1 **(1)**	10 **(8)**	5 **(2)**	26 **(2)**
Other	8 **(4)**	6 **(5)**	10 **(6)**	14 **(6)**	3 **(2)**	5 **(4)**	6 **(2)**	52 **(4)**
Total^b^	183	121	176	248	189	133	318	1,368

*^a^Numbers in bolded parentheses are percentages of each behavior group out of total interactions that the elephant exhibited, not always 100% due to rounding*.

*^b^Behavior totals, adjusted for time on test differ significantly among animals (*p* < 0.0001)*.

*^c^Totals differ significantly across all behaviors (*p* < 0.0001) or across four most common behaviors (trunk out through seeking, *p* < 0.0001)*.

The most common behavior groups used to initiate interactions were “trunk to human,” which occurred in 561 instances and “trunk out,” which was exhibited 432 times. “Trunk-out,” “trunk to human,” and “seeking” behaviors were the predominant behaviors exhibited both overall and in interactions directed toward guides (see Tables 3 and 5). “Trunk to human” and “trunk-out” behaviors also predominated for interactions directed toward volunteers and tourists, yet, seeking behaviors varied greatly in frequency depending on the individual elephant (see Tables 6 and 7). Differences in frequency of behavior groups exhibited were still significant when only the four most common behavior groups (“trunk to human,” “trunk out,” “seeking,” “trunk to object”) were analyzed (*p* < 0.0001).

**Table 5:** **Behavior group vs. elephant (directed toward guides only)**.

	Sally	Nandi	Thandi	Keisha	Mashudu	Thato	Shungu	Total^c^
Trunk out	37 **(32^a^)**	44 **(39)**	43 **(27)**	54 **(33)**	32 **(33)**	18 **(24)**	67 **(36)**	295 **(33)**
Trunk to human	60 **(52)**	35 **(31)**	54 **(34)**	65 **(40)**	32 **(33)**	23 **(30)**	45 **(24)**	314 **(35)**
Trunk to object	9 **(8)**	9 **(8)**	14 **(9)**	2 **(1)**	3 **(3)**	9 **(12)**	8 **(4)**	54 **(6)**
Seeking	3 **(3)**	18 **(16)**	34 **(21)**	26 **(16)**	27 **(28)**	17 **(22)**	57 **(31)**	182 **(20)**
Prolonged contact	0 **(0)**	0 **(0)**	5 **(3)**	3 **(2)**	1 **(1)**	6 **(8)**	3 **(2)**	18 **(2)**
Other	6 **(5)**	6 **(5)**	9 **(6)**	12 **(7)**	1 **(1)**	3 **(4)**	6 **(3)**	43 **(5)**
Total^b^	115	112	159	162	96	76	186	906

*^a^Numbers in bolded parentheses are percentages of each behavior group out of total interactions that the elephant exhibited toward guides, not always 100% due to rounding*.

*^b^Geometric mean across behavior groups directed toward guides differs significantly among elephants (*p* = 0.0008). However, the main effect of elephant is hard to interpret given that it involves non-trivial averaging across behavior groups*.

*^c^Totals directed toward guides differ significantly across all behaviors (*p* < 0.0001) or across four most common behaviors (trunk out through seeking, *p* < 0.0001)*.

**Table 6:** **Behavior group vs. elephant (directed toward volunteers only)**.

	Sally	Nandi	Thandi	Keisha	Mashudu	Thato	Shungu	Total^c^
Trunk out	0 **(0^a^)**	0 **(0)**	3 **(30)**	4 **(15)**	21 **(30)**	4 **(14)**	20 **(24)**	52 **(22)**
Trunk to human	7 **(88)**	2 **(50)**	5 **(50)**	18 **(69)**	35 **(50)**	17 **(59)**	47 **(55)**	131 **(56)**
Trunk to object	0 **(0)**	2 **(50)**	1 **(10)**	3 **(12)**	6 **(9)**	1 **(3)**	2 **(2)**	15 **(6)**
Seeking	0 **(0)**	0 **(0)**	1 **(10)**	1 **(4)**	6 **(9)**	4 **(14)**	14 **(16)**	26 **(11)**
Prolonged contact	0 **(0)**	0 **(0)**	0 **(0)**	0 **(0)**	0 **(0)**	2 **(7)**	2 **(2)**	4 **(2)**
Other	1 **(13)**	0 **(0)**	0 **(0)**	0 **(0)**	2 **(3)**	1 **(3)**	0 **(0)**	4 **(2)**
Total^b^	8	4	10	26	70	29	85	232

*^a^Numbers in bolded parentheses are percentages of each behavior group out of total interactions that the elephant exhibited toward volunteers, not always 100% due to rounding*.

*^b^Geometric mean across behavior groups directed toward volunteers differ significantly among elephants (*p* < 0.0001). However, the main effect of elephant is hard to interpret given that it involves non-trivial averaging across behavior groups*.

*^c^Totals directed toward volunteers differ significantly across all behaviors (*p* < 0.0001) and across four most common behaviors (trunk out through seeking, *p* < 0.0001)*.

**Table 7:** **Behavior group vs. elephant (directed toward tourists only)**.

	Sally	Nandi	Thandi	Keisha	Mashudu	Thato	Shungu	Total^c^
Trunk out	12 **(20^a^)**	3 **(60)**	5 **(71)**	29 **(48)**	8 **(35)**	9 **(32)**	19 **(40)**	85 **(37)**
Trunk to human	39 **(65)**	1 **(20)**	1 **(14)**	23 **(38)**	15 **(65)**	15 **(54)**	22 **(47)**	116 **(50)**
Trunk to object	8 **(13)**	1 **(20)**	0 **(0)**	3 **(5)**	0 **(0)**	0 **(0)**	3 **(6)**	15 **(7)**
Seeking	0 **(0)**	0 **(0)**	0 **(0)**	1 **(2)**	0 **(0)**	1 **(4)**	3 **(6)**	5 **(2)**
Prolonged contact	0 **(0)**	0 **(0)**	0 **(0)**	2 **(3)**	0 **(0)**	2 **(7)**	0 **(0)**	4 **(2)**
Other	1 **(2)**	0 **(0)**	1 **(14)**	2 **(3)**	0 **(0)**	1 **(4)**	0 **(0)**	5 **(2)**
Total^b^	60	5	7	60	23	28	47	230

*^a^Numbers in bolded parentheses are percentages of each behavior group out of total interactions that the elephant exhibited toward tourists, not always 100% due to rounding*.

*^b^Geometric mean across behavior groups directed toward tourists differ significantly among elephants (*p* < 0.0001). However, the main effect of elephant is hard to interpret given that it involves non-trivial averaging across behavior groups*.

*^c^Totals directed toward tourists differ significantly across all behaviors (*p* < 0.0001) or across four most common behaviors (trunk out through seeking, *p* < 0.0001)*.

### Preferences toward a Specific Category of Human

**Figure 2:**
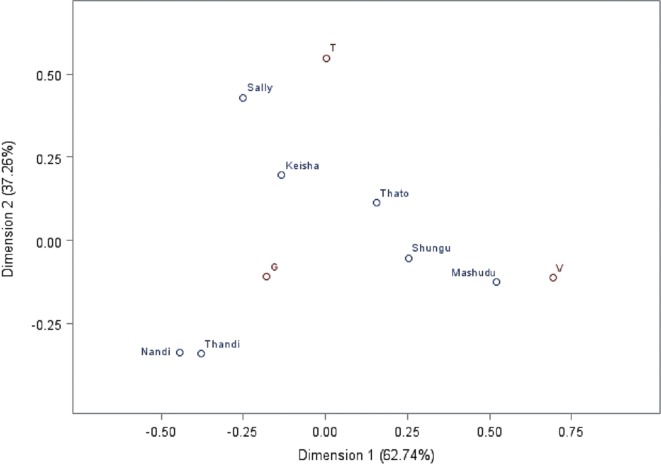
**Elephant by category of human canonical correspondence analysis (CCA)**. The figure shows an elephant by category of human CCA. Elephants are represented by name, and category of human is shown with G for guide, V for volunteer, and T for tourist. This plot shows the best two-dimensional representation of the relationships between individual elephants and category of humans. Dimension 1 and Dimension 2 are selected to explain as much of the dependence between these two variables as possible, with Dimension 1 explaining the most dependence, and Dimension 2 the second most. In this plot, preference is functionally indicated by both how close pairs of elephant-category points are to each other and their position respective to the origin of the plot.

Individual elephants varied in the types of humans with whom they chose to initiate interactions (*p* < 0.0001). Certain elephants interacted almost exclusively with guides, while several other elephants interacted more with volunteers or tourists relative to other individuals in the herd (see Figures 1 and 2).

Overall, the seven elephants preferred to interact with guides rather than with tourists and volunteers (*p* < 0.0001, see Table 3). There was not a significant difference in the total interactions exhibited toward tourists as opposed to volunteers (*p* = 0.997).

Seeking out behaviors accounted for 20% of all behaviors toward guides, compared to volunteers (11%) and tourists (2%).

### Preferences toward a Specific Individual

**Figure 3:**
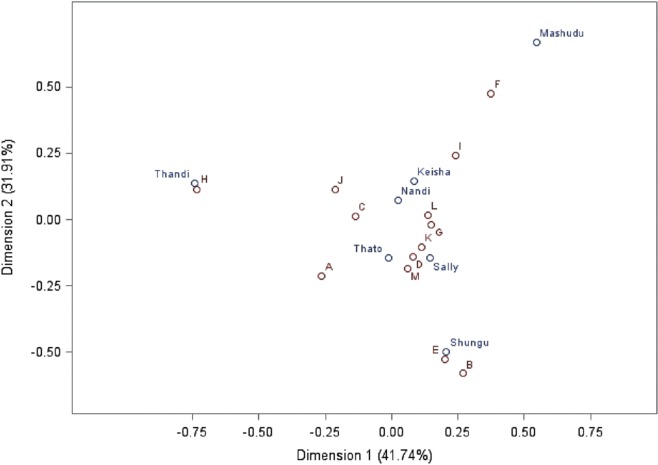
**Elephant by guide canonical correspondence analysis (CCA)**. The figure shows an elephant by guide CCA. Elephants are represented by name, and guides are represented with letter codes A–M. For information on how to read this plot, see Figure 2. In this plot, preference is functionally indicated by both how close pairs of elephant–guide points are to each other and their position respective to the origin of the plot. Important elephant–guide pairs shown on this plot are Shungu with E, Mashudu with F, and Thandi with H.

As a group, the elephants interacted preferentially with some guides over others (*p* < 0.0001). Individual elephants did not differ significantly in the geometric mean of the number of interactions initiated toward all guides ($\chi_{6}^{2}=5.45$, *p* = 0.4881). However, certain elephants interacted preferentially with specific guides ($\chi_{66}^{2}=102.56$, *p* = 0.0026). The most significant of these elephant–guide pairings were Mashudu with guide F, Shungu with guide E, and Thandi with guide H (see Figure 3).

## Discussion

### Variation in Types and Frequencies of Interactions

Shungu, the most subordinate elephant in the herd, interacted more than any other elephant, especially with guides and volunteers. As a male, he is not incorporated into the herd’s core social group (Sally, Nandi, Thandi, and Thato—see Table 1), and he is also lower in status than the other young male, Mashudu. Keisha and Mashudu, the other two elephants not involved in the core social group, initiated the next two highest numbers of interactions total. The combined number of interactions initiated by the four core elephants (613) was less than the combined number of interactions initiated by the three non-core elephants (755). Since African elephants are extremely social animals, one potential future hypothesis is that these elephants may seek out humans to get the social interactions that they lack with their conspecifics.

Another possible direction would be to consider whether these elephants are experiencing something similar to the “safe haven” effect, where an animal views a human as a source of safety, and is less susceptible to stress factors in the presence of that human (1). In certain cases, animals have been shown to feel safer around humans than around conspecifics (1, 2). The elephants initiating high numbers of interactions may be using humans in general as a type of safe haven to avoid any negative interactions with conspecifics, especially from the high-ranking females in the herd.

“Trunk out” behaviors were performed at the highest percentage toward tourists, and “trunk to human” behaviors were performed more toward tourists and volunteers than toward guides. As a group, the elephants consistently exhibited “trunk to human” behaviors at a higher frequency than “trunk out” behaviors. “Trunk out” and “trunk to human” are both short, exploratory behaviors. When tourists are feeding the elephants from across the barrier, the two behaviors exhibited are “trunk out” and “trunk to human.” Although no data are collected during feeding, these behaviors performed away from the barrier may, in certain cases, be indirectly food motivated. “Trunk out” and “trunk to human” account for the highest percentage of interactions toward tourists, yet also are the most frequently exhibited behaviors for volunteers and guides (see Table 3). It is likely that there is an exploratory component associated with these behaviors that may also help explain the higher percentages for tourists and volunteers.

Conversely, “seeking” behaviors were performed at the lowest percentage toward tourists (2%, see Table 3). “Seeking” behaviors were the third most common behaviors exhibited toward guides and volunteers, but a much lower fourth most common behavior exhibited toward tourists. “Seeking” behaviors were directed at the highest percentage toward guides (20%), then volunteers (11%) and last tourists (2%). Since “seeking” behaviors seem to indicate a higher level of commitment from the elephant initiating the interaction, these elephants may be less committed to interacting with tourists. The higher percentage of “seeking” behaviors directed toward volunteers and the even higher percentage directed toward guides support these behaviors as bonding behaviors. Since “seeking” behaviors put the elephant in close proximity to the human target, these behaviors also provide an opportunity for further interactions that require close proximity, such as “trunk out” and “trunk to human.” This may help account for the fact that “trunk to human” and “trunk out” behaviors exceed “seeking” behaviors in numbers, even for guides.

Although “seeking” behaviors occurred more often than “trunk to object” behaviors overall, there were some notable exceptions for categories of human and for individual elephants. For interactions directed toward guides, “seeking” behaviors exceeded “trunk to object” behaviors both overall and for every elephant except Sally (see Table 5). Sally performed “seeking out” behaviors the least of all elephants (three interactions total, all toward a guide), potentially indicating that seeking out human interaction is not as necessary for the elephant with the strongest social position in the herd.

“Seeking” behaviors exceeded “trunk to object” behaviors overall for volunteers as well; however, individually, this order was actually only seen for Shungu and Thato, the two most subordinate elephants (see Table 6). “Seeking” behaviors initiated by Shungu alone could account for why “seeking” behaviors outweighed “trunk to object” behaviors toward volunteers overall. The frequency of “seeking” behaviors exhibited by Shungu and Thato toward volunteers could support the aforementioned “safe haven” hypothesis.

Tourists were the only category of human where “trunk to object” behaviors exceeded “seeking” behaviors overall (see Table 7). One potential explanation for this is that tourists are the only type of human who regularly bring bags out to the field. The elephants may be curious about the contents, and especially, the possibility that a tourist may have food in his or her bag. Additionally, groups of tourists are often brought to the elephants, or a guide may issue a command for an elephant to move near a group of tourists. Since human-initiated behaviors and behaviors in response to commands were not looked at in this study, it may be that the elephants were indeed often in close proximity to tourists, yet rarely initiated this proximity. Only Thato exhibited “seeking” behaviors slightly more frequently than “trunk to object” behaviors toward tourists (1–0).

The existence of specific temperaments within the herd may be supported by the different ranges in frequencies of behavior groups exhibited by each individual animal, as described by Mills (39) for horses. These different behavior profiles have been shown to carry predictive weight (24) and may be an additional useful tool and a potential area for further investigation when considering the management of small groups of elephants managed in free contact. However, there were similarities in behavior patterns across the elephants as well, indicating similar overall temperaments for this group of elephants who have lived together and watched each other over a relatively long period of time.

### Preferences toward a Specific Category of Human

As a whole, Sally’s herd showed a preference for interacting with guides over volunteers and tourists. This was expected given the amount of time the guides spend directly handling and interacting with the elephants. Every elephant in the herd directed more than 50% of behaviors toward guides (see Tables 5–7; Figure 1). This seems to be an indication that there may be the potential for bonding between elephants and guides.

Nandi and Thandi, the only related elephants in the herd, rarely interacted with tourists or volunteers. They share a close bond as mother and daughter, and also entertain strong relationships with Sally, the matriarch, and Thato, the youngest female. Nandi and Thandi’s occupation with intraspecific relationships within the herd may be a contributing factor to their disinclination to interact with volunteers and tourists.

The four lower-ranking elephants interacted more with volunteers than the three higher-ranking elephants. Shungu and Mashudu, the two male elephants (and not a part of the core group) interacted the most with volunteers. Since volunteers do not generally feed the elephants (like tourists), and there is not a long-term relationship built with the elephants (like guides), there is a lack of food-based or bond-based motivation to interact with volunteers. Therefore, the data showing that lower ranked elephants interact more often with volunteers potentially support the aforementioned “safe-haven” hypothesis.

### Preferences toward a Specific Individual

Considering that HABs have been most often reported in large mammals (8), and that there is evidence for HABs between Asian elephants and mahouts (17), the potential for HABs between African elephants and guides is a relevant issue. Physical contact is considered to be important in the development of bonds (1), and these elephants have regular physical contact with the guides through the elephant-initiated interactions studied, as well as through guide-initiated interactions, training, and general care.

This study makes a convincing argument for HABs between three of the African elephants and guides at KEP. The most important elephant–guide pairings, based on numbers of interactions, were Shungu-Guide E, Mashudu-Guide F, and Thandi-Guide H. These findings matched with three of the main anecdotal elephant–guide “friendships” recognized by staff and volunteers at KEP. These three elephants also performed numerically more “seeking out” behaviors toward guides than the other elephants did. These “seeking out” behaviors are widely considered to be one indicator of HARs and bonds (1, 5–7). Importantly, the exhibition of “seeking out” behaviors does not relate to the total number of interactions, meaning that the high number of “seeking out” behaviors cannot be attributed to more interactions overall.

In addition to looking at elephant–guide bonds experimentally through interaction data, guides’ perspectives of the elephants were used to determine reciprocity of these bonds. AERU has conducted surveys on the guides’ opinions of the elephants, including guide preferences for specific elephants and information on elephant personality traits (28). The three guides with identified bonds with a specific elephant all ranked their respective bonded elephants as either their first or second favorite. They also described that particular elephant as responding to their commands either best or second best. Each of these guides ranked his bonded elephant in the top three (out of seven) for measures of confidence, curiosity, and activity. Although these particular guides found their particular elephants to be more responsive to their commands, they also viewed the elephants participating in these bonds as bolder, more exploratory animals. The scores by guides involved in bonds are not necessarily representative of the overall views of the guides toward the elephants involved in bonds. That is to say, bonded guides held more favorable views of the elephant they shared a bond with than non-bonded guides did of those same elephants. This is evidence that these elephant–guide bonds are indeed reciprocal, and not solely based on elephant preference.

Human–animal bonds may foster a safer environment for both the human and animal involved in the bond. HABs have been shown to generate operational and affective benefits (8) and positive interactions between humans and animals can also lead to an increase in affiliative behaviors between conspecifics (3, 4). These findings have special implications for animals managed in free contact. Ensuring the safety of both the humans and animals in this type of setting is a fundamental challenge, so any findings that contribute to the maintenance of a safe free contact environment should not be overlooked.

## Ethics Statement

The UC Davis IACUC office found the study exempt from approval on an Animal Care and Use Protocol because of its observational nature. Permission to observe the elephants was granted by the management of Knysna Elephant Park and the African Elephant Research Unit in Knysna, Western Cape, South Africa.

## Author Contributions

Study design; manuscript drafting and editing: ZR, CP, DY, and LH. Data collection and analysis: ZR.

## Conflict of Interest Statement

The authors declare that the research was conducted in the absence of any commercial or financial relationships that could be construed as a potential conflict of interest.
