# Supplementary material for: Elephant-Initiated Interactions with Humans: Individual Differences and Specific Preferences in Captive African Elephants (Loxodonta africana)
Source: Front Vet Sci. 2017 Apr 28;4:60. doi: 10.3389/fvets.2017.00060 (PMC5408011; doi:10.3389/fvets.2017.00060)
Supplement: Supplementary file 1 [file data_sheet_1.pdf]

Date	Time	Elephant	Behavior	Behavior group	Type of human	Specification
6/27/15	11:56	Shungu	Trunk out	Trunk out	G	A
6/27/15	11:56	Shungu	Trunk out	Trunk out	T	
6/27/15	12:25	Thandi	Head-lean	Prolonged contact	G	H
6/27/15	13:00	Sally	Trunk out	Trunk out	G	D
6/27/15	13:03	Sally	Trunk to body	Trunk to human	T	
6/27/15	13:04	Sally	Trunk to foot	Trunk to human	G	Z
6/27/15	13:14	Keisha	Trunk to body	Trunk to human	T	
6/27/15	13:22	Thato	Trunk to hand	Trunk to human	T	
6/27/15	13:36	Keisha	Trunk out	Trunk out	G	G
6/28/15	11:25	Thato	Trunk to body	Trunk to human	T	
6/28/15	11:25	Thato	Trunk to foot	Trunk to human	T	
6/28/15	11:27	Thato	Trunk out	Trunk out	T	
6/28/15	11:27	Thato	Trunk to foot	Trunk to human	T	
6/28/15	11:29	Thato	Trunk out	Trunk out	T	
6/28/15	11:30	Thato	Trunk to hand	Trunk to human	T	
6/28/15	11:30	Thato	Trunk out	Trunk out	T	
6/28/15	11:32	Thato	Trunk to hand	Trunk to human	T	
6/28/15	11:39	Thandi	Trunk out	Trunk out	T	
6/28/15	11:39	Thandi	Trunk out	Trunk out	T	
6/28/15	11:39	Thandi	Trunk out	Trunk out	T	
6/28/15	11:48	Keisha	Trunk out	Trunk out	G	D
6/28/15	11:48	Keisha	Trunk to foot	Trunk to human	G	D
6/28/15	11:54	Shungu	Trunk out	Trunk out	G	E
6/28/15	11:54	Thandi	Approach	Seeking out	G	D
6/28/15	11:54	Nandi	Trunk to hand	Trunk to human	G	H
6/28/15	11:54	Nandi	Trunk to body	Trunk to human	G	H
6/28/15	11:58	Shungu	Approach	Seeking out	G	E
6/28/15	11:58	Shungu	Trunk to hand	Trunk to human	G	E

6/28/15	12:01	Keisha	Trunk to hand	Trunk to human	V	
6/28/15	12:01	Keisha	Trunk out	Trunk out	G	D
6/28/15	12:01	Keisha	Trunk to body prolonged	Prolonged contact	T	
6/28/15	12:06	Shungu	Trunk out	Trunk out	G	H
6/28/15	12:06	Shungu	Approach	Seeking out	G	H
6/28/15	12:06	Shungu	Head-lean	Prolonged contact	G	H
6/28/15	12:07	Shungu	Trunk to leg	Trunk to human	V	
6/28/15	12:07	Shungu	Trunk to hand	Trunk to human	V	
6/28/15	12:09	Shungu	Trunk to hand	Trunk to human	T	
6/28/15	12:11	Shungu	Trunk to hand	Trunk to human	T	
6/28/15	12:13	Nandi	Approach	Seeking out	G	D
6/28/15	12:13	Nandi	Trunk out	Trunk out	G	D
6/28/15	12:13	Nandi	Trunk to bullhook	Trunk to object	G	D
6/28/15	12:16	Thandi	Approach	Seeking out	G	H
6/28/15	12:16	Thandi	Head-lean	Prolonged contact	G	H
6/28/15	12:16	Thandi	Trunk to bullhook	Trunk to object	G	H
6/28/15	12:16	Thandi	Trunk to hand	Trunk to human	G	H
6/28/15	12:16	Thandi	Trunk out	Trunk out	G	H
6/28/15	12:16	Thandi	Trunk to hand	Trunk to human	G	H
6/28/15	12:17	Nandi	Trunk to leg	Trunk to human	G	J
6/28/15	12:18	Shungu	Trunk to hand	Trunk to human	T	
6/28/15	12:19	Shungu	Trunk to hand	Trunk to human	G	E
6/28/15	12:19	Shungu	Trunk to hand	Trunk to human	V	
6/28/15	12:19	Shungu	Trunk to arm	Trunk to human	V	
6/28/15	12:20	Shungu	Trunk to hand	Trunk to human	V	
6/28/15	14:55	Shungu	Trunk to hand	Trunk to human	G	E
6/28/15	14:55	Shungu	Trunk to body	Trunk to human	V	
6/28/15	14:55	Shungu	Trunk to personal item	Trunk to object	V	
6/28/15	14:57	Thandi	Trunk to bullhook	Trunk to object	G	J

6/28/15	15:01	Thandi	Trunk to hand	Trunk to human	G	H
6/28/15	15:29	Nandi	Approach	Seeking out	G	K
6/28/15	15:29	Nandi	Ears out	Other	G	K
6/28/15	15:31	Keisha	Trunk to body	Trunk to human	G	K
6/29/15	11:27	Keisha	Trunk out	Trunk out	G	C
6/29/15	11:27	Keisha	Trunk out	Trunk out	G	H
6/29/15	11:27	Sally	Trunk to body	Trunk to human	G	L
6/29/15	11:27	Sally	Trunk to foot	Trunk to human	G	C
6/29/15	11:34	Sally	Trunk to hand	Trunk to human	G	C
6/29/15	11:34	Sally	Trunk to body	Trunk to human	G	C
6/29/15	11:42	Thandi	Trunk to foot	Trunk to human	V	
6/29/15	11:42	Thandi	Trunk to leg	Trunk to human	V	
6/29/15	11:42	Thandi	Trunk out	Trunk out	V	
6/29/15	11:45	Keisha	Trunk to leg	Trunk to human	G	K
6/29/15	11:45	Thato	Trunk to hand	Trunk to human	V	
6/29/15	11:48	Keisha	Trunk to body	Trunk to human	V	
6/29/15	11:52	Nandi	Trunk out	Trunk out	G	G
6/29/15	11:53	Shungu	Approach	Seeking out	G	K
6/29/15	11:56	Thandi	Trunk out	Trunk out	G	H
6/29/15	11:59	Shungu	Head-lean	Prolonged contact	G	L
6/29/15	12:04	Keisha	Trunk to hand	Trunk to human	V	
6/29/15	12:05	Nandi	Trunk to bullhook	Trunk to object	G	H
6/30/15	9:44	Mashudu	Trunk to hand	Trunk to human	V	
6/30/15	9:49	Keisha	Trunk to leg	Trunk to human	G	F
6/30/15	9:50	Keisha	Trunk out	Trunk out	G	G
6/30/15	10:04	Keisha	Approach	Seeking out	T	
6/30/15	10:25	Keisha	Trunk to hand	Trunk to human	G	F
6/30/15	10:25	Keisha	Trunk to body	Trunk to human	G	F
6/30/15	10:25	Keisha	Trunk out	Trunk out	G	F

6/30/15	11:27	Keisha	Trunk out	Trunk out	G	H
6/30/15	11:28	Thato	Trunk to leg	Trunk to human	V	
6/30/15	11:33	Keisha	Trunk out	Trunk out	G	K
6/30/15	11:34	Shungu	Trunk out	Trunk out	T	
6/30/15	11:47	Keisha	Trunk to body	Trunk to human	V	
6/30/15	11:47	Keisha	Trunk to hand	Trunk to human	V	
6/30/15	14:27	Mashudu	Trunk to body	Trunk to human	G	H
6/30/15	15:55	Thandi	Head-to	Other	G	A
6/30/15	15:55	Thandi	Trunk to foot	Trunk to human	G	A
6/30/15	15:56	Thandi	Trunk out	Trunk out	G	M
6/30/15	15:56	Mashudu	Trunk to personal item	Trunk to object	V	
6/30/15	15:57	Thandi	Trunk to hand	Trunk to human	G	M
6/30/15	15:57	Keisha	Head-to	Other	G	M
6/30/15	15:57	Keisha	Head-lean	Prolonged contact	G	M
6/30/15	16:04	Sally	Trunk out	Trunk out	G	A
7/1/15	8:59	Keisha	Approach	Seeking out	G	H
7/1/15	9:22	Thandi	Approach	Seeking out	G	D
7/1/15	9:22	Thandi	Trunk to body	Trunk to human	G	D
7/1/15	9:54	Keisha	Trunk out	Trunk out	G	F
7/1/15	9:54	Keisha	Trunk to bullhook	Trunk to object	G	F
7/1/15	9:54	Keisha	Trunk to body	Trunk to human	T	
7/1/15	10:15	Shungu	Follow	Seeking out	G	F
7/1/15	10:15	Shungu	Trunk to body	Trunk to human	G	F
7/1/15	10:26	Keisha	Trunk to body	Trunk to human	G	K
7/1/15	10:26	Thato	Approach	Seeking out	G	A
7/1/15	10:29	Keisha	Trunk out	Trunk out	G	K
7/1/15	10:36	Thandi	Approach	Seeking out	G	L
7/1/15	10:54	Shungu	Approach	Seeking out	G	H
7/1/15	11:21	Sally	Trunk to body	Trunk to human	G	L

7/1/15	11:21	Sally	Trunk to hand	Trunk to human	G	L
7/1/15	12:55	Shungu	Trunk to personal item	Trunk to object	T	
7/1/15	13:20	Keisha	Approach	Seeking out	G	F
7/1/15	13:45	Keisha	Approach	Seeking out	G	F
7/1/15	14:01	Keisha	Trunk to body	Trunk to human	G	F
7/1/15	14:01	Keisha	Trunk to body	Trunk to human	T	
7/1/15	14:01	Keisha	Trunk out	Trunk out	G	F
7/1/15	14:06	Sally	Trunk to body	Trunk to human	G	K
7/1/15	14:06	Sally	Trunk to arm	Trunk to human	G	K
7/1/15	14:08	Sally	Trunk to foot	Trunk to human	T	
7/1/15	14:08	Sally	Trunk to foot	Trunk to human	T	
7/1/15	14:44	Nandi	Face	Other	G	F
7/1/15	14:44	Nandi	Trunk to bullhook	Trunk to object	G	F
7/1/15	14:47	Shungu	Approach	Seeking out	V	
7/1/15	14:52	Shungu	Trunk out	Trunk out	G	K
7/1/15	15:00	Thandi	Face	Other	G	H
7/1/15	15:00	Shungu	Approach	Seeking out	T	
7/1/15	15:00	Shungu	Trunk out	Trunk out	T	
7/2/15	10:48	Mashudu	Trunk out	Trunk out	G	E
7/2/15	10:53	Keisha	Trunk to body	Trunk to human	G	D
7/2/15	11:17	Thandi	Approach	Seeking out	G	F
7/2/15	11:32	Sally	Trunk to leg	Trunk to human	G	G
7/2/15	11:45	Shungu	Approach	Seeking out	G	E
7/2/15	11:45	Shungu	Trunk out	Trunk out	G	E
7/2/15	14:33	Mashudu	Approach	Seeking out	G	F
7/2/15	14:33	Mashudu	Trunk to hand	Trunk to human	G	F
7/2/15	14:34	Mashudu	Trunk to hand	Trunk to human	G	F
7/2/15	14:34	Mashudu	Trunk to leg	Trunk to human	G	F
7/2/15	14:34	Shungu	Trunk to hand	Trunk to human	G	E

7/2/15	14:34	Shungu	Trunk to bullhook	Trunk to object	G	E
7/2/15	15:00	Shungu	Trunk to hand	Trunk to human	G	L
7/2/15	15:17	Thato	Trunk to hand	Trunk to human	T	
7/2/15	15:22	Nandi	Trunk to leg	Trunk to human	G	D
7/2/15	15:26	Keisha	Approach	Seeking out	G	L
7/2/15	15:26	Keisha	Head-lean	Prolonged contact	G	L
7/2/15	15:59	Thato	Trunk out	Trunk out	G	D
7/2/15	15:59	Keisha	Trunk to hand	Trunk to human	G	L
7/2/15	16:02	Keisha	Trunk out	Trunk out	G	L
7/2/15	16:02	Keisha	Head-to	Other	G	L
7/2/15	16:02	Keisha	Trunk to hand	Trunk to human	G	L
7/2/15	16:09	Keisha	Approach	Seeking out	G	L
7/2/15	16:10	Keisha	Follow	Seeking out	G	L
7/2/15	16:11	Keisha	Follow	Seeking out	G	L
7/2/15	16:11	Keisha	Trunk to hand	Trunk to human	V	NR
7/2/15	16:13	Shungu	Approach	Seeking out	G	K
7/2/15	16:13	Shungu	Approach	Seeking out	G	F
7/2/15	16:14	Shungu	Trunk to hand	Trunk to human	T	
7/2/15	16:20	Shungu	Approach	Seeking out	V	
7/2/15	16:23	Shungu	Trunk out	Trunk out	G	B
7/2/15	16:24	Shungu	Approach	Seeking out	V	
7/2/15	16:24	Shungu	Follow	Seeking out	V	
7/2/15	16:24	Shungu	Trunk out	Trunk out	V	
7/2/15	16:24	Shungu	Trunk to hand	Trunk to human	V	
7/2/15	16:25	Shungu	Trunk out	Trunk out	T	
7/2/15	16:26	Sally	Trunk to personal item	Trunk to object	T	
7/2/15	16:27	Sally	Trunk to leg	Trunk to human	G	H
7/2/15	16:28	Sally	Trunk to leg	Trunk to human	G	H
7/4/15	10:43	Mashudu	Approach	Seeking out	G	E

7/4/15	10:57	Keisha	Trunk to hand	Trunk to human	V	
7/4/15	10:57	Keisha	Trunk to personal item	Trunk to object	V	
7/4/15	10:57	Mashudu	Trunk out	Trunk out	G	E
7/4/15	11:04	Sally	Trunk to body	Trunk to human	T	
7/4/15	11:04	Sally	Trunk to body	Trunk to human	T	
7/4/15	11:05	Thandi	Approach	Seeking out	G	C
7/4/15	11:09	Sally	Trunk out	Trunk out	G	L
7/4/15	11:10	Sally	Approach	Seeking out	G	L
7/4/15	11:13	Thandi	Ears out	Other	G	F
7/4/15	11:21	Shungu	Trunk out	Trunk out	G	A
7/4/15	11:21	Shungu	Trunk out	Trunk out	G	B
7/4/15	11:21	Shungu	Head-to	Other	G	A
7/4/15	11:28	Shungu	Trunk to bullhook	Trunk to object	G	Z
7/4/15	11:28	Shungu	Trunk out	Trunk out	G	Z
7/4/15	11:29	Shungu	Trunk to leg	Trunk to human	G	Z
7/4/15	11:29	Shungu	Trunk to body	Trunk to human	G	Z
7/4/15	11:29	Shungu	Trunk out	Trunk out	G	Z
7/4/15	11:31	Thandi	Trunk to bullhook	Trunk to object	G	M
7/4/15	11:33	Shungu	Approach	Seeking out	G	E
7/4/15	11:33	Shungu	Follow	Seeking out	G	E
7/4/15	12:59	Keisha	Trunk to arm	Trunk to human	G	E
7/4/15	12:59	Keisha	Trunk out	Trunk out	G	E
7/4/15	14:04	Shungu	Trunk to hand	Trunk to human	T	
7/5/15	12:07	Shungu	Approach	Seeking out	V	
7/5/15	12:07	Shungu	Trunk to hand	Trunk to human	V	
7/5/15	12:07	Shungu	Trunk to body	Trunk to human	V	
7/5/15	12:24	Sally	Trunk to foot	Trunk to human	G	Z
7/5/15	12:24	Sally	Trunk out	Trunk out	G	Z
7/5/15	12:28	Shungu	Trunk out	Trunk out	V	

7/5/15	12:40	Sally	Trunk to personal item	Trunk to object	T	
7/5/15	14:50	Shungu	Trunk to hand	Trunk to human	V	
7/5/15	14:54	Shungu	Trunk out	Trunk out	G	E
7/5/15	15:02	Nandi	Trunk to leg	Trunk to human	G	F
7/5/15	15:29	Nandi	Trunk out	Trunk out	G	E
7/5/15	15:56	Shungu	Approach	Seeking out	G	A
7/5/15	15:56	Shungu	Ear flap	Other	G	A
7/6/15	9:05	Sally	Trunk to hand	Trunk to human	G	A
7/6/15	9:05	Sally	Trunk out	Trunk out	G	A
7/6/15	9:05	Sally	Trunk to body	Trunk to human	G	A
7/6/15	9:10	Nandi	Trunk out	Trunk out	G	H
7/6/15	10:34	Shungu	Trunk to hand	Trunk to human	G	K
7/6/15	10:35	Shungu	Trunk out	Trunk out	G	K
7/6/15	10:36	Thato	Approach	Seeking out	G	G
7/6/15	10:37	Keisha	Trunk to body	Trunk to human	G	K
7/6/15	10:37	Keisha	Trunk to hand	Trunk to human	G	K
7/6/15	10:39	Keisha	Trunk to hand	Trunk to human	G	K
7/6/15	10:40	Sally	Trunk out	Trunk out	G	E
7/6/15	10:41	Keisha	Trunk to hand	Trunk to human	G	K
7/6/15	10:49	Shungu	Approach	Seeking out	G	G
7/6/15	11:00	Shungu	Trunk out	Trunk out	G	G
7/6/15	11:03	Mashudu	Trunk to body	Trunk to human	T	
7/6/15	11:07	Shungu	Trunk to foot	Trunk to human	G	C
7/6/15	11:29	Sally	Trunk to leg	Trunk to human	G	G
7/6/15	11:51	Shungu	Trunk to hand	Trunk to human	V	
7/6/15	11:59	Shungu	Trunk out	Trunk out	G	H
7/6/15	12:00	Sally	Face	Other	G	G
7/6/15	12:39	Keisha	Trunk to leg	Trunk to human	T	
7/6/15	12:39	Keisha	Trunk out	Trunk out	T	

7/6/15	14:43	Mashudu	Trunk out	Trunk out	V	
7/6/15	15:06	Keisha	Trunk to hand	Trunk to human	G	E
7/6/15	15:57	Thandi	Trunk out	Trunk out	G	H
7/6/15	15:57	Thandi	Trunk to leg	Trunk to human	G	H
7/6/15	16:26	Shungu	Trunk to personal item	Trunk to object	T	
7/6/15	16:26	Shungu	Approach	Seeking out	G	E
7/6/15	16:27	Thato	Trunk out	Trunk out	G	C
7/6/15	16:28	Shungu	Trunk to body	Trunk to human	T	
7/7/15	9:04	Thandi	Approach	Seeking out	G	L
7/7/15	12:32	Keisha	Approach	Seeking out	G	G
7/7/15	12:33	Thandi	Trunk out	Trunk out	G	H
7/7/15	12:33	Thandi	Trunk to body	Trunk to human	G	H
7/7/15	12:33	Keisha	Trunk out	Trunk out	T	
7/7/15	12:34	Keisha	Trunk out	Trunk out	T	
7/7/15	12:37	Keisha	Trunk out	Trunk out	T	
7/7/15	12:38	Sally	Trunk to leg	Trunk to human	G	Z
7/7/15	12:58	Keisha	Approach	Seeking out	G	L
7/7/15	13:26	Keisha	Trunk out	Trunk out	T	
7/7/15	13:29	Keisha	Trunk out	Trunk out	G	D
7/7/15	13:38	Shungu	Trunk to body	Trunk to human	T	
7/7/15	13:53	Shungu	Trunk out	Trunk out	G	C
7/7/15	13:53	Shungu	Trunk out	Trunk out	G	E
7/7/15	13:59	Thandi	Trunk to hand	Trunk to human	G	G
7/7/15	14:02	Thandi	Trunk to leg	Trunk to human	G	C
7/7/15	14:28	Thandi	Trunk out	Trunk out	G	D
7/7/15	14:29	Keisha	Trunk to arm	Trunk to human	G	C
7/7/15	14:29	Nandi	Trunk to bullhook	Trunk to object	G	B
7/7/15	14:30	Keisha	Trunk to hand	Trunk to human	G	B
7/7/15	14:30	Keisha	Trunk to hand	Trunk to human	T	

7/7/15	15:32	Shungu	Trunk out	Trunk out	V	
7/7/15	15:35	Thandi	Trunk out	Trunk out	G	H
7/7/15	15:35	Thandi	Trunk to body	Trunk to human	G	H
7/7/15	15:36	Shungu	Trunk out	Trunk out	G	K
7/7/15	15:36	Keisha	Trunk out	Trunk out	G	H
7/7/15	15:39	Thato	Trunk out	Trunk out	G	H
7/7/15	15:49	Sally	Trunk to leg	Trunk to human	G	D
7/7/15	15:49	Sally	Trunk to body	Trunk to human	T	
7/7/15	16:27	Nandi	Trunk out	Trunk out	G	B
7/7/15	16:27	Nandi	Trunk out	Trunk out	G	H
7/7/15	16:28	Sally	Trunk out	Trunk out	G	H
7/7/15	16:32	Shungu	Trunk out	Trunk out	G	E
7/8/15	11:24	Thandi	Approach	Seeking out	G	H
7/8/15	11:24	Thandi	Trunk out	Trunk out	G	H
7/8/15	11:30	Thato	Trunk to leg	Trunk to human	G	E
7/8/15	11:30	Thato	Trunk to hand	Trunk to human	G	E
7/8/15	11:30	Thato	Trunk out	Trunk out	G	E
7/8/15	11:30	Shungu	Approach	Seeking out	G	E
7/8/15	11:30	Shungu	Trunk to hand	Trunk to human	G	E
7/8/15	11:31	Shungu	Face	Other	G	E
7/8/15	11:31	Shungu	Trunk to hand	Trunk to human	G	E
7/8/15	11:32	Shungu	Trunk to body	Trunk to human	G	E
7/8/15	11:59	Shungu	Trunk out	Trunk out	G	F
7/8/15	12:03	Shungu	Trunk out	Trunk out	G	F
7/8/15	12:08	Thandi	Trunk out	Trunk out	T	
7/8/15	12:08	Thandi	Trunk out	Trunk out	G	F
7/8/15	12:15	Thandi	Trunk to leg	Trunk to human	G	F
7/8/15	12:16	Sally	Head-to	Other	G	F
7/8/15	12:19	Thato	Trunk to body	Trunk to human	G	F

7/8/15	12:58	Sally	Trunk out	Trunk out	G	F
7/8/15	12:59	Sally	Trunk to leg	Trunk to human	G	F
7/8/15	13:53	Shungu	Approach	Seeking out	G	E
7/8/15	13:53	Shungu	Trunk out	Trunk out	G	E
7/8/15	13:55	Shungu	Face	Other	G	M
7/8/15	15:50	Nandi	Trunk to body	Trunk to human	G	K
7/8/15	15:55	Thandi	Trunk to leg	Trunk to human	G	K
7/8/15	16:02	Sally	Trunk to body	Trunk to human	T	
7/8/15	16:24	Nandi	Trunk to leg	Trunk to human	G	C
7/8/15	16:27	Shungu	Approach	Seeking out	G	F
7/8/15	16:27	Shungu	Trunk to hand	Trunk to human	G	F
7/8/15	16:28	Keisha	Trunk to body	Trunk to human	G	H
7/9/15	10:54	Keisha	Trunk to body	Trunk to human	T	
7/9/15	11:20	Mashudu	Head-to	Other	G	F
7/9/15	11:32	Shungu	Trunk to hand	Trunk to human	T	
7/9/15	11:33	Thandi	Trunk out	Trunk out	G	C
7/9/15	11:34	Keisha	Face	Other	G	C
7/9/15	11:34	Keisha	Trunk to leg	Trunk to human	G	C
7/9/15	11:39	Sally	Trunk to hand	Trunk to human	G	NR
7/9/15	12:06	Sally	Trunk to hand	Trunk to human	G	A
7/9/15	14:01	Shungu	Approach	Seeking out	G	D
7/9/15	14:29	Nandi	Trunk to bullhook	Trunk to object	G	H
7/9/15	14:29	Nandi	Trunk to body	Trunk to human	G	H
7/9/15	15:10	Shungu	Trunk out	Trunk out	G	M
7/9/15	15:16	Keisha	Trunk to hand	Trunk to human	G	A
7/9/15	15:16	Keisha	Trunk out	Trunk out	T	
7/9/15	15:21	Thato	Trunk to hand	Trunk to human	G	D
7/9/15	15:39	Sally	Trunk to leg	Trunk to human	G	F
7/9/15	15:39	Sally	Trunk to body	Trunk to human	G	F

7/9/15	15:49	Thandi	Approach	Seeking out	G	H
7/9/15	15:49	Thandi	Trunk to body	Trunk to human	G	H
7/9/15	15:52	Mashudu	Trunk out	Trunk out	V	
7/9/15	16:12	Thandi	Approach	Seeking out	V	
7/12/15	10:21	Thandi	Approach	Seeking out	G	H
7/12/15	10:21	Thandi	Trunk to body	Trunk to human	G	H
7/12/15	11:21	Mashudu	Trunk out	Trunk out	V	
7/12/15	11:52	Thandi	Approach	Seeking out	G	F
7/12/15	14:20	Keisha	Trunk to leg	Trunk to human	G	E
7/12/15	14:20	Sally	Trunk out	Trunk out	G	E
7/12/15	14:23	Shungu	Approach	Seeking out	V	
7/12/15	14:23	Shungu	Trunk out	Trunk out	V	
7/12/15	14:26	Thandi	Trunk to body	Trunk to human	G	H
7/12/15	14:27	Mashudu	Trunk to hand	Trunk to human	T	
7/12/15	14:27	Mashudu	Trunk to hand	Trunk to human	T	
7/12/15	14:33	Shungu	Trunk to leg	Trunk to human	G	Z
7/12/15	14:34	Keisha	Trunk out	Trunk out	T	
7/12/15	14:34	Keisha	Trunk to leg	Trunk to human	T	
7/12/15	14:34	Keisha	Trunk out	Trunk out	T	
7/12/15	14:35	Keisha	Trunk to hand prolonged	Prolonged contact	T	
7/12/15	14:35	Keisha	Trunk to hand	Trunk to human	T	
7/12/15	14:35	Keisha	Trunk to bullhook	Trunk to object	G	L
7/12/15	14:36	Keisha	Trunk out	Trunk out	G	H
7/12/15	14:36	Keisha	Head-to	Other	T	
7/12/15	14:39	Mashudu	Trunk to hand	Trunk to human	V	
7/12/15	14:39	Mashudu	Trunk to foot	Trunk to human	G	Z
7/12/15	14:40	Keisha	Trunk to body	Trunk to human	V	
7/12/15	14:40	Keisha	Trunk to arm	Trunk to human	V	
7/12/15	14:41	Keisha	Trunk to body	Trunk to human	T	

7/12/15	14:41	Keisha	Trunk out	Trunk out	T	
7/12/15	14:41	Keisha	Trunk to foot	Trunk to human	G	Z
7/12/15	14:41	Keisha	Trunk to leg	Trunk to human	G	Z
7/12/15	14:41	Keisha	Trunk to body	Trunk to human	G	Z
7/12/15	14:41	Thato	Trunk to hand	Trunk to human	G	H
7/12/15	14:41	Thato	Trunk to bullhook	Trunk to object	G	H
7/12/15	14:44	Keisha	Trunk to hand	Trunk to human	T	
7/12/15	14:44	Sally	Trunk to body	Trunk to human	V	
7/12/15	14:44	Sally	Trunk to body	Trunk to human	T	
7/12/15	14:46	Sally	Trunk to foot	Trunk to human	T	
7/12/15	14:47	Thandi	Trunk to foot	Trunk to human	G	H
7/12/15	14:48	Thato	Trunk to leg	Trunk to human	G	L
7/12/15	14:48	Keisha	Trunk out	Trunk out	T	
7/12/15	14:49	Mashudu	Trunk out	Trunk out	T	
7/12/15	14:49	Mashudu	Trunk to hand	Trunk to human	G	L
7/12/15	14:49	Mashudu	Trunk to leg	Trunk to human	G	L
7/12/15	14:49	Mashudu	Trunk to body	Trunk to human	G	L
7/12/15	15:28	Sally	Trunk out	Trunk out	G	K
7/12/15	15:29	Nandi	Trunk out	Trunk out	G	H
7/12/15	15:30	Nandi	Trunk to body	Trunk to human	T	
7/12/15	15:42	Thandi	Trunk out	Trunk out	G	L
7/12/15	15:59	Mashudu	Trunk to leg	Trunk to human	T	
7/12/15	15:59	Mashudu	Trunk to foot	Trunk to human	T	
7/13/15	11:25	Shungu	Approach	Seeking out	G	A
7/13/15	11:26	Nandi	Trunk out	Trunk out	G	L
7/13/15	11:33	Mashudu	Trunk to body	Trunk to human	G	F
7/13/15	11:40	Thandi	Approach	Seeking out	G	F
7/13/15	11:51	Thandi	Trunk out	Trunk out	G	F
7/13/15	12:33	Nandi	Head-to	Other	G	F

7/13/15	12:33	Nandi	Trunk to body	Trunk to human	G	F
7/13/15	12:38	Nandi	Trunk out	Trunk out	G	C
7/13/15	12:49	Shungu	Approach	Seeking out	G	G
7/13/15	12:50	Thato	Trunk to bullhook	Trunk to object	G	F
7/13/15	12:51	Mashudu	Trunk to leg	Trunk to human	G	D
7/13/15	12:57	Mashudu	Approach	Seeking out	G	F
7/13/15	12:57	Mashudu	Trunk to leg	Trunk to human	G	D
7/13/15	12:59	Keisha	Trunk to hand	Trunk to human	G	C
7/13/15	13:09	Thato	Approach	Seeking out	V	
7/13/15	14:49	Mashudu	Approach	Seeking out	G	F
7/13/15	14:49	Mashudu	Trunk to body	Trunk to human	G	F
7/13/15	15:38	Thandi	Trunk to personal item	Trunk to object	G	M
7/13/15	15:53	Mashudu	Approach	Seeking out	G	E
7/13/15	15:53	Thato	Trunk out	Trunk out	G	C
7/13/15	15:55	Sally	Trunk to leg	Trunk to human	G	F
7/13/15	15:56	Keisha	Trunk out	Trunk out	G	A
7/13/15	15:58	Nandi	Trunk out	Trunk out	G	L
7/13/15	16:23	Nandi	Trunk to leg	Trunk to human	G	B
7/13/15	16:23	Nandi	Trunk to leg	Trunk to human	G	D
7/13/15	16:25	Sally	Trunk to foot	Trunk to human	G	A
7/13/15	16:25	Nandi	Trunk to bullhook	Trunk to object	G	M
7/13/15	16:28	Sally	Trunk out	Trunk out	T	
7/13/15	16:30	Mashudu	Approach	Seeking out	G	G
7/14/15	10:55	Mashudu	Trunk out	Trunk out	V	
7/14/15	10:56	Sally	Trunk to body	Trunk to human	G	A
7/14/15	10:57	Thandi	Trunk to bullhook	Trunk to object	G	Z
7/14/15	10:57	Nandi	Trunk out	Trunk out	G	D
7/14/15	11:05	Thandi	Trunk to leg	Trunk to human	G	C
7/14/15	11:05	Thandi	Approach	Seeking out	G	C

7/14/15	11:05	Thandi	Approach	Seeking out	G	E
7/14/15	11:05	Thandi	Trunk to body	Trunk to human	G	E
7/14/15	11:25	Thato	Trunk to body	Trunk to human	G	M
7/14/15	12:02	Thato	Trunk to body	Trunk to human	G	F
7/14/15	12:20	Shungu	Trunk out	Trunk out	G	E
7/14/15	14:26	Mashudu	Approach	Seeking out	G	D
7/14/15	14:56	Sally	Trunk to body	Trunk to human	G	L
7/14/15	15:00	Mashudu	Trunk to body	Trunk to human	G	F
7/14/15	15:01	Shungu	Trunk out	Trunk out	T	
7/14/15	15:05	Nandi	Trunk to leg	Trunk to human	G	F
7/14/15	15:18	Nandi	Approach	Seeking out	G	C
7/14/15	15:18	Nandi	Trunk to foot	Trunk to human	G	C
7/14/15	15:19	Shungu	Approach	Seeking out	G	D
7/14/15	15:19	Shungu	Trunk out	Trunk out	G	D
7/14/15	15:19	Shungu	Trunk out	Trunk out	G	M
7/14/15	15:38	Keisha	Trunk out	Trunk out	G	K
7/14/15	16:11	Shungu	Approach	Seeking out	G	E
7/14/15	16:11	Shungu	Trunk to body	Trunk to human	G	E
7/14/15	16:15	Shungu	Approach	Seeking out	G	E
7/14/15	16:41	Mashudu	Approach	Seeking out	V	
7/14/15	16:41	Mashudu	Trunk out	Trunk out	V	
7/14/15	16:41	Mashudu	Approach	Seeking out	V	
7/14/15	16:41	Mashudu	Trunk to body	Trunk to human	V	
7/15/15	10:54	Shungu	Trunk to body	Trunk to human	G	M
7/15/15	10:59	Shungu	Trunk to arm	Trunk to human	G	M
7/15/15	12:35	Thandi	Trunk to hand	Trunk to human	G	A
7/15/15	12:57	Sally	Trunk out	Trunk out	G	Z
7/15/15	13:03	Sally	Trunk to leg	Trunk to human	G	Z
7/15/15	13:35	Keisha	Trunk out	Trunk out	T	

7/15/15	13:40	Thato	Trunk to hand	Trunk to human	G	D
7/15/15	14:27	Keisha	Trunk out	Trunk out	T	
7/15/15	14:27	Mashudu	Approach	Seeking out	G	M
7/15/15	14:27	Mashudu	Trunk to hand	Trunk to human	G	M
7/15/15	14:33	Nandi	Trunk out	Trunk out	G	E
7/15/15	14:38	Sally	Trunk out	Trunk out	G	E
7/15/15	14:42	Shungu	Approach	Seeking out	V	
7/15/15	14:42	Shungu	Trunk to hand	Trunk to human	V	
7/15/15	14:42	Shungu	Trunk to hand	Trunk to human	T	
7/15/15	14:43	Shungu	Trunk to personal item	Trunk to object	G	B
7/15/15	14:43	Thato	Approach	Seeking out	G	B
7/15/15	14:43	Thato	Trunk to personal item	Trunk to object	G	B
7/15/15	14:55	Keisha	Trunk out	Trunk out	T	
7/15/15	14:55	Keisha	Trunk out	Trunk out	G	H
7/15/15	14:56	Keisha	Trunk to body	Trunk to human	T	
7/15/15	14:57	Sally	Trunk to foot	Trunk to human	T	
7/15/15	15:13	Thato	Trunk to leg	Trunk to human	T	
7/16/15	9:33	Shungu	Trunk to hand	Trunk to human	V	
7/16/15	9:33	Shungu	Trunk out	Trunk out	V	
7/16/15	9:33	Shungu	Approach	Seeking out	V	
7/16/15	9:33	Shungu	Trunk to hand	Trunk to human	V	
7/16/15	9:33	Shungu	Trunk out	Trunk out	V	
7/16/15	9:33	Shungu	Trunk to hand	Trunk to human	V	
7/16/15	9:33	Shungu	Trunk out	Trunk out	V	
7/16/15	9:34	Shungu	Follow	Seeking out	V	
7/16/15	9:52	Shungu	Trunk to hand	Trunk to human	V	
7/16/15	10:17	Keisha	Trunk to hand	Trunk to human	G	E
7/16/15	10:24	Shungu	Trunk to leg	Trunk to human	G	C
7/16/15	10:25	Thato	Approach	Seeking out	V	

7/16/15	10:25	Thato	Trunk to body	Trunk to human	V	
7/16/15	10:25	Thato	Trunk to hand	Trunk to human	G	C
7/16/15	10:37	Keisha	Trunk to foot	Trunk to human	G	E
7/16/15	10:47	Thato	Approach	Seeking out	G	H
7/16/15	10:47	Thato	Trunk to bullhook	Trunk to object	G	H
7/16/15	10:47	Thato	Head-lean	Prolonged contact	G	H
7/16/15	10:48	Thato	Trunk to arm prolonged	Prolonged contact	V	
7/16/15	10:48	Thato	Trunk to leg	Trunk to human	V	
7/16/15	10:48	Thato	Trunk to head	Trunk to human	V	
7/16/15	10:48	Thato	Trunk to body	Trunk to human	V	
7/16/15	10:48	Thato	Trunk to head	Trunk to human	V	
7/16/15	10:48	Thato	Trunk to leg	Trunk to human	V	
7/16/15	10:49	Thato	Trunk to hand	Trunk to human	V	
7/16/15	10:49	Thato	Trunk to leg	Trunk to human	V	
7/16/15	10:49	Thato	Trunk to leg	Trunk to human	V	
7/16/15	10:50	Thato	Trunk to leg	Trunk to human	V	
7/16/15	10:50	Thato	Trunk to hand	Trunk to human	V	
7/16/15	10:52	Thandi	Approach	Seeking out	G	H
7/16/15	10:52	Thandi	Trunk out	Trunk out	G	H
7/16/15	11:01	Keisha	Approach	Seeking out	V	
7/16/15	11:01	Keisha	Trunk to hand	Trunk to human	V	
7/16/15	11:01	Keisha	Trunk to body	Trunk to human	V	
7/16/15	11:01	Keisha	Trunk out	Trunk out	G	L
7/16/15	11:02	Keisha	Trunk to hand	Trunk to human	G	M
7/16/15	11:11	Thato	Approach	Seeking out	G	G
7/16/15	11:21	Shungu	Approach	Seeking out	G	E
7/16/15	11:21	Shungu	Trunk to body	Trunk to human	G	E
7/16/15	11:21	Shungu	Trunk out	Trunk out	G	E
7/16/15	11:21	Shungu	Face	Other	G	E

7/16/15	11:21	Shungu	Follow	Seeking out	G	E
7/16/15	11:21	Shungu	Trunk to hand	Trunk to human	V	
7/16/15	13:31	Nandi	Trunk to body	Trunk to human	G	E
7/16/15	13:31	Nandi	Trunk out	Trunk out	G	E
7/16/15	13:42	Nandi	Trunk out	Trunk out	T	
7/16/15	13:52	Nandi	Trunk to body	Trunk to human	G	L
7/16/15	13:53	Sally	Trunk out	Trunk out	G	L
7/16/15	13:54	Sally	Tail hit	Other	T	
7/16/15	14:17	Shungu	Trunk out	Trunk out	G	D
7/16/15	14:18	Mashudu	Trunk to hand	Trunk to human	V	
7/16/15	14:28	Keisha	Face	Other	G	E
7/16/15	14:28	Keisha	Trunk to body	Trunk to human	G	E
7/16/15	14:30	Keisha	Trunk out	Trunk out	G	E
7/16/15	14:38	Sally	Trunk to body	Trunk to human	G	E
7/16/15	15:30	Shungu	Trunk out	Trunk out	V	
7/16/15	15:49	Thato	Approach	Seeking out	G	E
7/16/15	15:49	Thato	Trunk out	Trunk out	G	E
7/17/15	15:26	Shungu	Approach	Seeking out	G	D
7/17/15	15:26	Shungu	Trunk out	Trunk out	G	D
7/17/15	15:26	Shungu	Trunk to leg	Trunk to human	G	D
7/17/15	15:26	Mashudu	Trunk out	Trunk out	V	
7/17/15	15:27	Thato	Trunk to body	Trunk to human	G	H
7/17/15	15:32	Keisha	Trunk out	Trunk out	T	
7/17/15	15:57	Keisha	Trunk out	Trunk out	G	E
7/17/15	15:57	Keisha	Trunk out	Trunk out	T	
7/17/15	16:01	Mashudu	Trunk to hand	Trunk to human	V	
7/17/15	16:01	Mashudu	Trunk to hand	Trunk to human	V	
7/17/15	16:01	Mashudu	Trunk out	Trunk out	V	
7/17/15	16:18	Shungu	Trunk to leg	Trunk to human	G	F

7/17/15	16:23	Mashudu	Approach	Seeking out	G	F
7/17/15	16:23	Mashudu	Trunk to hand	Trunk to human	G	F
7/17/15	16:23	Mashudu	Trunk to arm	Trunk to human	G	F
7/17/15	16:23	Mashudu	Trunk out	Trunk out	G	F
7/17/15	16:24	Mashudu	Approach	Seeking out	V	
7/18/15	8:35	Mashudu	Trunk out	Trunk out	V	
7/18/15	8:35	Mashudu	Trunk to arm	Trunk to human	G	K
7/18/15	8:43	Sally	Head-to	Other	V	
7/18/15	8:43	Sally	Trunk to hand	Trunk to human	V	
7/20/15	9:06	Mashudu	Trunk out	Trunk out	V	
7/20/15	9:10	Thandi	Trunk to foot	Trunk to human	G	L
7/20/15	9:12	Thandi	Trunk out	Trunk out	G	A
7/20/15	9:12	Thandi	Approach	Seeking out	G	A
7/20/15	9:12	Thandi	Head-lean	Prolonged contact	G	A
7/20/15	13:17	Thandi	Trunk to body	Trunk to human	G	K
7/20/15	13:37	Thato	Approach	Seeking out	G	B
7/21/15	10:58	Thandi	Approach	Seeking out	G	H
7/21/15	10:58	Thandi	Trunk to leg	Trunk to human	G	H
7/21/15	11:03	Thato	Push	Other	G	D
7/21/15	11:19	Mashudu	Trunk to body	Trunk to human	G	F
7/21/15	11:19	Mashudu	Trunk to bullhook	Trunk to object	G	F
7/21/15	11:19	Mashudu	Trunk to leg	Trunk to human	G	F
7/21/15	11:19	Mashudu	Trunk to leg	Trunk to human	G	F
7/21/15	11:21	Mashudu	Trunk out	Trunk out	G	F
7/21/15	11:21	Mashudu	Approach	Seeking out	V	
7/21/15	11:24	Keisha	Trunk out	Trunk out	G	F
7/21/15	11:55	Shungu	Trunk out	Trunk out	T	
7/21/15	11:55	Shungu	Trunk out	Trunk out	T	
7/22/15	13:11	Keisha	Trunk to foot	Trunk to human	T	

7/22/15	13:17	Thato	Trunk to hand	Trunk to human	T	
7/22/15	13:17	Thato	Trunk to arm	Trunk to human	T	
7/22/15	13:21	Nandi	Trunk out	Trunk out	G	F
7/22/15	13:36	Thandi	Trunk out	Trunk out	G	D
7/22/15	13:57	Keisha	Trunk out	Trunk out	G	E
7/22/15	13:57	Keisha	Trunk to body	Trunk to human	G	E
7/22/15	13:59	Keisha	Approach	Seeking out	G	F
7/22/15	13:59	Keisha	Trunk to body	Trunk to human	G	F
7/22/15	14:26	Nandi	Trunk out	Trunk out	G	F
7/22/15	14:29	Sally	Trunk to hand	Trunk to human	T	
7/22/15	14:29	Sally	Trunk to body	Trunk to human	T	
7/22/15	14:35	Sally	Trunk to body	Trunk to human	G	E
7/22/15	14:35	Sally	Trunk to leg	Trunk to human	G	E
7/22/15	14:35	Sally	Trunk to body	Trunk to human	G	E
7/22/15	14:40	Mashudu	Trunk to hand	Trunk to human	T	
7/22/15	14:40	Mashudu	Trunk to hand	Trunk to human	T	
7/22/15	14:40	Mashudu	Trunk to leg	Trunk to human	T	
7/22/15	14:44	Thato	Trunk to hand	Trunk to human	V	
7/22/15	15:19	Nandi	Approach	Seeking out	G	M
7/22/15	15:19	Nandi	Trunk out	Trunk out	G	M
7/22/15	16:16	Mashudu	Trunk to body	Trunk to human	G	G
7/22/15	16:16	Thato	Trunk to hand	Trunk to human	G	B
7/22/15	16:18	Shungu	Approach	Seeking out	G	I
7/22/15	16:18	Sally	Ears out	Other	G	B
7/22/15	16:19	Mashudu	Trunk out	Trunk out	G	F
7/22/15	16:20	Mashudu	Trunk to body	Trunk to human	T	
7/22/15	16:20	Mashudu	Trunk to leg	Trunk to human	G	F
7/22/15	16:20	Mashudu	Trunk to hand	Trunk to human	G	F
7/22/15	16:21	Mashudu	Trunk to hand	Trunk to human	G	F

7/22/15	16:21	Shungu	Approach	Seeking out	V	
7/22/15	16:21	Shungu	Trunk out	Trunk out	V	
7/22/15	16:21	Shungu	Approach	Seeking out	G	B
7/23/15	10:33	Mashudu	Trunk out	Trunk out	G	F
7/23/15	10:36	Thato	Trunk out	Trunk out	V	
7/23/15	11:12	Thandi	Approach	Seeking out	G	E
7/23/15	11:18	Keisha	Approach	Seeking out	G	C
7/23/15	11:22	Thato	Approach	Seeking out	G	A
7/23/15	11:24	Thato	Branch throw	Other	G	A
7/23/15	11:32	Mashudu	Trunk out	Trunk out	V	
7/23/15	12:11	Sally	Trunk to body	Trunk to human	G	Z
7/23/15	12:11	Sally	Trunk to leg	Trunk to human	T	
7/23/15	12:24	Shungu	Trunk to hand	Trunk to human	V	
7/23/15	14:21	Keisha	Trunk out	Trunk out	V	
7/23/15	14:24	Nandi	Trunk to hand	Trunk to human	G	M
7/23/15	14:25	Thandi	Trunk out	Trunk out	G	M
7/23/15	14:26	Thato	Trunk to hand	Trunk to human	V	
7/23/15	14:29	Nandi	Trunk out	Trunk out	G	B
7/23/15	14:38	Shungu	Trunk out	Trunk out	G	E
7/23/15	15:00	Shungu	Trunk out	Trunk out	V	
7/24/15	12:03	Thato	Approach	Seeking out	T	
7/24/15	12:16	Thandi	Head-to	Other	G	G
7/24/15	12:34	Mashudu	Trunk out	Trunk out	V	
7/24/15	13:00	Shungu	Trunk out	Trunk out	V	
7/24/15	13:38	Keisha	Trunk out	Trunk out	G	C
7/24/15	13:53	Shungu	Trunk out	Trunk out	G	M
7/24/15	13:53	Shungu	Trunk to body	Trunk to human	G	M
7/24/15	13:53	Shungu	Trunk out	Trunk out	G	M
7/24/15	14:23	Shungu	Trunk to leg	Trunk to human	G	B

7/24/15	14:24	Thato	Trunk out	Trunk out	T	
7/24/15	15:02	Sally	Trunk to leg	Trunk to human	G	C
7/24/15	16:04	Sally	Trunk to foot	Trunk to human	T	
7/24/15	16:06	Sally	Trunk to leg	Trunk to human	T	
7/24/15	16:06	Sally	Trunk to body	Trunk to human	T	
7/24/15	16:06	Sally	Trunk to leg	Trunk to human	T	
7/24/15	16:09	Sally	Trunk to leg	Trunk to human	T	
7/26/15	8:53	Mashudu	Trunk out	Trunk out	V	
7/26/15	9:32	Thandi	Trunk out	Trunk out	T	
7/26/15	9:50	Thandi	Trunk to body	Trunk to human	G	E
7/26/15	9:50	Thandi	Trunk to bullhook	Trunk to object	G	E
7/26/15	10:06	Thato	Approach	Seeking out	G	C
7/26/15	10:12	Thandi	Trunk to head	Trunk to human	G	C
7/26/15	10:37	Sally	Trunk out	Trunk out	G	M
7/26/15	10:37	Sally	Trunk to leg	Trunk to human	G	H
7/26/15	10:51	Shungu	Trunk out	Trunk out	G	K
7/26/15	10:51	Keisha	Trunk out	Trunk out	G	E
7/27/15	8:48	Sally	Trunk to personal item	Trunk to object	T	
7/27/15	8:48	Sally	Trunk to leg	Trunk to human	T	
7/27/15	8:50	Thandi	Trunk out	Trunk out	V	
7/27/15	8:50	Thandi	Trunk out	Trunk out	G	E
7/27/15	8:54	Thandi	Approach	Seeking out	G	A
7/27/15	9:17	Mashudu	Trunk to hand	Trunk to human	V	
7/27/15	9:17	Mashudu	Trunk to leg	Trunk to human	V	
7/27/15	13:56	Sally	Trunk out	Trunk out	G	I
7/27/15	14:45	Keisha	Trunk out	Trunk out	G	Y
7/27/15	15:19	Thato	Trunk out	Trunk out	V	
7/27/15	15:25	Shungu	Approach	Seeking out	V	
7/27/15	15:25	Shungu	Trunk to hand	Trunk to human	V	

7/27/15	15:25	Shungu	Trunk to bullhook	Trunk to object	G	D
7/27/15	15:52	Sally	Trunk out	Trunk out	G	L
7/27/15	15:52	Keisha	Trunk to body	Trunk to human	G	M
7/27/15	15:56	Sally	Trunk out	Trunk out	T	
7/27/15	16:26	Nandi	Trunk out	Trunk out	G	I
7/27/15	16:46	Mashudu	Trunk out	Trunk out	V	
7/27/15	16:46	Mashudu	Trunk to hand	Trunk to human	V	
7/27/15	16:46	Mashudu	Trunk to body	Trunk to human	V	
7/27/15	16:46	Mashudu	Trunk to hand	Trunk to human	V	
7/27/15	16:46	Mashudu	Trunk to hand	Trunk to human	V	
7/29/15	10:15	Keisha	Approach	Seeking out	G	K
7/29/15	10:31	Nandi	Trunk to leg	Trunk to human	G	L
7/29/15	10:39	Keisha	Approach	Seeking out	G	F
7/29/15	10:39	Mashudu	Approach	Seeking out	G	C
7/29/15	11:02	Sally	Trunk to body	Trunk to human	G	F
7/29/15	11:24	Nandi	Approach	Seeking out	G	A
7/29/15	11:24	Thandi	Trunk out	Trunk out	G	H
7/29/15	11:24	Thandi	Trunk flick	Other	G	H
7/29/15	11:24	Thandi	Trunk to body	Trunk to human	G	H
7/29/15	11:24	Thandi	Follow	Seeking out	G	H
7/29/15	11:24	Thandi	Trunk out	Trunk out	G	H
7/29/15	11:27	Sally	Trunk out	Trunk out	T	
7/29/15	11:27	Sally	Trunk to hand	Trunk to human	G	I
7/29/15	11:27	Sally	Trunk to personal item	Trunk to object	T	
7/29/15	11:28	Sally	Trunk out	Trunk out	T	
7/29/15	11:33	Sally	Trunk out	Trunk out	G	I
7/29/15	12:00	Shungu	Trunk to hand	Trunk to human	V	
7/29/15	12:11	Shungu	Approach	Seeking out	G	J
7/29/15	13:23	Keisha	Approach	Seeking out	G	I

7/29/15	14:20	Shungu	Approach	Seeking out	G	F
7/29/15	14:20	Shungu	Trunk to foot	Trunk to human	G	F
7/29/15	14:20	Shungu	Trunk to hand	Trunk to human	V	
7/29/15	14:20	Shungu	Trunk to hand	Trunk to human	G	F
7/29/15	15:27	Thandi	Head-to	Other	G	J
7/29/15	15:38	Thandi	Face	Other	G	J
7/29/15	15:38	Thandi	Trunk out	Trunk out	G	J
7/29/15	15:41	Thato	Trunk out	Trunk out	G	C
7/29/15	15:43	Thato	Trunk to bullhook	Trunk to object	G	C
7/29/15	15:44	Sally	Approach	Seeking out	G	H
7/29/15	15:44	Sally	Trunk out	Trunk out	G	H
7/29/15	15:55	Thato	Approach	Seeking out	G	I
7/29/15	16:09	Mashudu	Approach	Seeking out	G	F
7/29/15	16:13	Mashudu	Approach	Seeking out	G	F
7/31/15	11:26	Keisha	Trunk out	Trunk out	T	
7/31/15	11:26	Shungu	Trunk out	Trunk out	G	I
7/31/15	11:34	Mashudu	Trunk out	Trunk out	G	I
7/31/15	11:42	Thato	Trunk to bullhook	Trunk to object	G	I
7/31/15	11:46	Shungu	Trunk to hand	Trunk to human	V	
7/31/15	11:48	Nandi	Approach	Seeking out	G	D
7/31/15	14:25	Shungu	Trunk to hand	Trunk to human	V	
7/31/15	14:25	Shungu	Trunk to hand	Trunk to human	V	
7/31/15	15:00	Sally	Trunk to hand	Trunk to human	V	
7/31/15	15:02	Sally	Trunk out	Trunk out	T	
7/31/15	15:08	Keisha	Trunk out	Trunk out	G	F
7/31/15	15:22	Shungu	Trunk out	Trunk out	V	
7/31/15	15:22	Shungu	Trunk out	Trunk out	G	Z
8/1/15	12:57	Sally	Trunk out	Trunk out	G	Z
8/1/15	12:57	Sally	Trunk to leg	Trunk to human	G	Z

8/1/15	12:57	Nandi	Trunk to hand	Trunk to human	G	H
8/1/15	13:00	Keisha	Trunk out	Trunk out	G	J
8/1/15	13:00	Keisha	Trunk to leg	Trunk to human	G	J
8/1/15	13:24	Keisha	Approach	Seeking out	G	L
8/1/15	13:58	Mashudu	Trunk out	Trunk out	V	
8/1/15	14:00	Sally	Trunk out	Trunk out	G	M
8/1/15	14:21	Shungu	Approach	Seeking out	T	
8/1/15	14:21	Shungu	Trunk to hand	Trunk to human	T	
8/1/15	14:22	Shungu	Trunk to leg	Trunk to human	T	
8/1/15	14:30	Sally	Trunk to bullhook	Trunk to object	G	F
8/1/15	14:48	Mashudu	Trunk to hand	Trunk to human	G	F
8/1/15	15:03	Sally	Trunk out	Trunk out	T	
8/1/15	15:12	Mashudu	Trunk to personal item	Trunk to object	G	Y
8/1/15	15:17	Keisha	Trunk to hand	Trunk to human	T	
8/1/15	15:52	Mashudu	Approach	Seeking out	G	I
8/1/15	16:07	Shungu	Approach	Seeking out	G	M
8/2/15	10:31	Mashudu	Trunk out	Trunk out	T	
8/2/15	10:31	Mashudu	Trunk to hand	Trunk to human	T	
8/2/15	10:32	Keisha	Trunk to leg	Trunk to human	T	
8/2/15	10:34	Thandi	Trunk out	Trunk out	G	F
8/2/15	10:36	Thandi	Trunk flick	Other	G	F
8/2/15	10:38	Thato	Trunk to hand	Trunk to human	G	M
8/2/15	10:42	Mashudu	Trunk out	Trunk out	G	F
8/2/15	10:42	Mashudu	Trunk to bullhook	Trunk to object	G	F
8/2/15	10:48	Sally	Trunk to body	Trunk to human	G	F
8/2/15	11:27	Shungu	Trunk to leg	Trunk to human	G	E
8/2/15	11:33	Sally	Trunk out	Trunk out	T	
8/2/15	11:33	Sally	Trunk out	Trunk out	T	
8/2/15	11:40	Sally	Trunk to personal item	Trunk to object	T	

8/2/15	11:58	Shungu	Trunk out	Trunk out	G	B
8/2/15	12:36	Thandi	Trunk to hand	Trunk to human	G	H
8/2/15	12:58	Keisha	Trunk out	Trunk out	G	F
8/2/15	13:05	Sally	Trunk to body	Trunk to human	G	F
8/3/15	13:54	Nandi	Trunk out	Trunk out	G	E
8/3/15	13:55	Sally	Trunk out	Trunk out	G	B
8/3/15	13:56	Sally	Trunk to arm	Trunk to human	G	B
8/3/15	14:24	Shungu	Approach	Seeking out	G	E
8/3/15	14:24	Shungu	Trunk out	Trunk out	G	E
8/3/15	15:27	Shungu	Approach	Seeking out	G	I
8/4/15	8:34	Shungu	Approach	Seeking out	G	E
8/4/15	9:28	Thandi	Trunk to body	Trunk to human	G	H
8/4/15	9:31	Thato	Trunk to body	Trunk to human	G	I
8/4/15	10:25	Shungu	Trunk to hand	Trunk to human	V	
8/4/15	10:25	Mashudu	Trunk to hand	Trunk to human	V	
8/4/15	10:25	Mashudu	Trunk to hand	Trunk to human	V	
8/4/15	10:35	Keisha	Trunk out	Trunk out	G	Y
8/4/15	10:55	Mashudu	Approach	Seeking out	G	H
8/4/15	10:55	Mashudu	Trunk out	Trunk out	G	H
8/4/15	13:07	Shungu	Follow	Seeking out	G	E
8/4/15	14:01	Mashudu	Trunk out	Trunk out	G	J
8/4/15	14:08	Sally	Trunk to bullhook	Trunk to object	G	B
8/4/15	14:31	Mashudu	Approach	Seeking out	G	I
8/4/15	14:43	Nandi	Approach	Seeking out	G	M
8/4/15	14:43	Nandi	Ears out	Other	G	M
8/4/15	14:53	Sally	Trunk out	Trunk out	G	D
8/4/15	15:07	Thandi	Approach	Seeking out	G	H
8/4/15	15:07	Thandi	Trunk out	Trunk out	G	H
8/5/15	12:55	Keisha	Trunk to body	Trunk to human	G	F

8/5/15	13:25	Keisha	Trunk out	Trunk out	G	I
8/5/15	13:26	Keisha	Follow	Seeking out	G	I
8/5/15	13:26	Keisha	Trunk out	Trunk out	G	I
8/5/15	15:57	Mashudu	Trunk to leg	Trunk to human	V	
8/5/15	15:57	Mashudu	Trunk to leg	Trunk to human	V	
8/5/15	15:57	Mashudu	Trunk to leg	Trunk to human	V	
8/5/15	15:57	Mashudu	Trunk out	Trunk out	V	
8/5/15	15:57	Mashudu	Trunk to personal item	Trunk to object	V	
8/5/15	16:03	Thandi	Trunk to hand	Trunk to human	V	
8/6/15	9:58	Shungu	Approach	Seeking out	G	F
8/6/15	10:00	Shungu	Approach	Seeking out	G	D
8/6/15	10:00	Keisha	Approach	Seeking out	G	F
8/6/15	10:00	Shungu	Approach	Seeking out	V	
8/6/15	10:55	Shungu	Trunk to leg	Trunk to human	T	
8/6/15	11:26	Keisha	Trunk out	Trunk out	G	Y
8/6/15	11:26	Keisha	Trunk to hand	Trunk to human	G	Y
8/6/15	11:28	Keisha	Trunk to hand	Trunk to human	G	I
8/6/15	11:47	Nandi	Trunk to bullhook	Trunk to object	G	I
8/6/15	11:56	Keisha	Trunk out	Trunk out	G	J
8/7/15	9:59	Shungu	Trunk out	Trunk out	G	B
8/7/15	10:27	Sally	Trunk to bullhook	Trunk to object	G	I
8/7/15	10:27	Sally	Trunk out	Trunk out	G	I
8/7/15	10:28	Mashudu	Trunk out	Trunk out	T	
8/7/15	10:28	Nandi	Trunk out	Trunk out	G	H
8/7/15	10:28	Mashudu	Trunk to hand	Trunk to human	T	
8/7/15	10:28	Sally	Trunk out	Trunk out	G	I
8/7/15	10:29	Keisha	Trunk to arm	Trunk to human	G	H
8/7/15	10:29	Keisha	Trunk to foot	Trunk to human	V	
8/7/15	10:30	Shungu	Trunk to personal item	Trunk to object	G	I

8/7/15	10:31	Keisha	Trunk out	Trunk out	G	L
8/7/15	10:32	Sally	Trunk to leg	Trunk to human	T	
8/7/15	10:32	Mashudu	Trunk to arm	Trunk to human	G	I
8/7/15	10:33	Sally	Trunk to foot	Trunk to human	T	
8/7/15	10:33	Sally	Trunk to bullhook	Trunk to object	G	J
8/7/15	10:36	Sally	Trunk to foot	Trunk to human	T	
8/7/15	10:36	Keisha	Trunk out	Trunk out	V	
8/7/15	10:37	Keisha	Trunk out	Trunk out	G	H
8/7/15	10:38	Thato	Approach	Seeking out	V	
8/7/15	10:38	Thato	Trunk out	Trunk out	V	
8/7/15	10:41	Nandi	Trunk to personal item	Trunk to object	T	
8/7/15	10:42	Keisha	Trunk out	Trunk out	G	L
8/7/15	10:42	Keisha	Trunk out	Trunk out	T	
8/7/15	10:42	Keisha	Trunk out	Trunk out	T	
8/7/15	10:44	Keisha	Trunk out	Trunk out	G	H
8/7/15	10:45	Keisha	Head-to	Other	G	H
8/7/15	10:45	Keisha	Trunk to foot	Trunk to human	T	
8/7/15	10:46	Keisha	Head-to	Other	G	H
8/7/15	10:46	Keisha	Trunk to leg	Trunk to human	G	H
8/7/15	10:46	Shungu	Approach	Seeking out	T	
8/7/15	10:46	Shungu	Approach	Seeking out	G	F
8/7/15	10:47	Keisha	Head-to	Other	G	H
8/7/15	10:47	Keisha	Approach	Seeking out	G	H
8/7/15	10:48	Shungu	Trunk out	Trunk out	T	
8/7/15	10:50	Keisha	Trunk out	Trunk out	T	
8/7/15	10:51	Keisha	Trunk to body	Trunk to human	G	L
8/8/15	9:56	Keisha	Trunk out	Trunk out	G	J
8/8/15	10:13	Thandi	Trunk to hand	Trunk to human	G	H
8/8/15	10:26	Shungu	Approach	Seeking out	G	L

8/8/15	10:26	Shungu	Trunk out	Trunk out	G	L
8/8/15	10:27	Shungu	Trunk out	Trunk out	G	H
8/8/15	10:27	Shungu	Trunk out	Trunk out	V	
8/8/15	11:17	Mashudu	Trunk out	Trunk out	V	
8/8/15	11:27	Mashudu	Trunk out	Trunk out	T	
8/8/15	11:30	Keisha	Trunk out	Trunk out	G	J
8/8/15	11:30	Keisha	Trunk to foot	Trunk to human	T	
8/8/15	11:30	Keisha	Trunk to hand	Trunk to human	T	
8/8/15	11:32	Shungu	Trunk to hand	Trunk to human	V	
8/8/15	11:39	Thandi	Approach	Seeking out	G	I
8/8/15	11:39	Thandi	Head-lean	Prolonged contact	G	I
8/8/15	11:40	Keisha	Trunk to leg	Trunk to human	T	
8/8/15	11:44	Sally	Trunk to foot	Trunk to human	T	
8/8/15	11:47	Shungu	Approach	Seeking out	G	A
8/8/15	11:47	Shungu	Trunk to hand	Trunk to human	T	
8/8/15	12:03	Mashudu	Trunk to hand	Trunk to human	T	
8/8/15	12:06	Mashudu	Trunk out	Trunk out	V	
8/8/15	12:06	Mashudu	Trunk to foot	Trunk to human	T	
8/8/15	12:14	Shungu	Approach	Seeking out	G	H
8/8/15	12:16	Shungu	Follow	Seeking out	G	H
8/8/15	12:18	Shungu	Follow	Seeking out	G	H
8/8/15	12:28	Keisha	Approach	Seeking out	G	L
8/8/15	12:28	Keisha	Trunk out	Trunk out	G	L
8/8/15	12:41	Sally	Trunk out	Trunk out	G	H
8/8/15	12:44	Thandi	Trunk to hand	Trunk to human	G	H
8/8/15	12:57	Sally	Trunk out	Trunk out	G	E
8/8/15	15:51	Thandi	Approach	Seeking out	G	H
8/8/15	15:51	Thandi	Trunk to body	Trunk to human	G	H
8/8/15	16:35	Nandi	Approach	Seeking out	G	H

8/8/15	16:35	Nandi	Trunk out	Trunk out	G	H
8/9/15	11:38	Thato	Approach	Seeking out	G	Y
8/9/15	11:38	Thato	Head-lean	Prolonged contact	G	Y
8/9/15	11:41	Thato	Trunk to leg	Trunk to human	G	E
8/9/15	11:41	Thato	Trunk out	Trunk out	T	
8/9/15	11:48	Sally	Trunk to leg	Trunk to human	T	
8/9/15	11:52	Thato	Trunk out	Trunk out	T	
8/9/15	12:11	Mashudu	Trunk to body	Trunk to human	T	
8/9/15	12:21	Thandi	Trunk to body	Trunk to human	G	F
8/9/15	12:21	Thandi	Trunk to bullhook	Trunk to object	G	F
8/9/15	12:34	Thato	Trunk to hand	Trunk to human	G	D
8/9/15	12:34	Thato	Trunk out	Trunk out	T	
8/9/15	12:34	Thato	Trunk out	Trunk out	G	D
8/9/15	12:37	Keisha	Trunk to body	Trunk to human	T	
8/9/15	12:39	Keisha	Trunk out	Trunk out	T	
8/9/15	12:42	Thato	Trunk to body prolonged	Prolonged contact	G	Y
8/9/15	12:42	Thandi	Trunk to leg	Trunk to human	G	Y
8/9/15	12:45	Thato	Approach	Seeking out	G	A
8/9/15	12:47	Thato	Approach	Seeking out	G	Y
8/9/15	12:47	Thato	Trunk to head	Trunk to human	G	Y
8/9/15	12:47	Thato	Trunk to body prolonged	Prolonged contact	G	Y
8/9/15	13:43	Thato	Trunk to leg	Trunk to human	T	
8/9/15	14:35	Mashudu	Trunk to hand	Trunk to human	V	
8/9/15	14:37	Mashudu	Trunk to hand	Trunk to human	G	I
8/9/15	14:42	Keisha	Trunk to leg	Trunk to human	G	I
8/9/15	14:42	Keisha	Trunk to body	Trunk to human	G	I
8/9/15	15:05	Nandi	Approach	Seeking out	G	F
8/9/15	15:06	Nandi	Trunk out	Trunk out	G	F
8/9/15	15:30	Shungu	Trunk out	Trunk out	G	E

8/9/15	15:31	Shungu	Trunk to leg	Trunk to human	T	
8/9/15	15:31	Shungu	Trunk to body	Trunk to human	T	
8/9/15	15:33	Sally	Trunk to leg	Trunk to human	T	
8/9/15	15:34	Shungu	Trunk to hand	Trunk to human	T	
8/9/15	15:43	Thato	Trunk to leg	Trunk to human	T	
8/9/15	15:43	Thato	Trunk to hand	Trunk to human	T	
8/9/15	15:55	Thandi	Trunk to cane	Trunk to object	G	D
8/9/15	15:56	Thandi	Trunk out	Trunk out	G	I
8/9/15	15:57	Nandi	Trunk to personal item	Trunk to object	V	
8/9/15	15:59	Mashudu	Trunk out	Trunk out	V	
8/9/15	15:59	Mashudu	Trunk to hand	Trunk to human	V	
8/9/15	16:17	Nandi	Trunk out	Trunk out	G	I
8/10/15	9:07	Mashudu	Trunk out	Trunk out	V	
8/10/15	9:35	Mashudu	Trunk out	Trunk out	G	F
8/10/15	9:35	Mashudu	Approach	Seeking out	G	F
8/10/15	9:43	Thandi	Trunk to leg	Trunk to human	G	F
8/10/15	9:45	Thato	Trunk out	Trunk out	G	F
8/10/15	9:47	Shungu	Approach	Seeking out	G	F
8/10/15	9:47	Shungu	Trunk out	Trunk out	G	F
8/10/15	9:47	Shungu	Trunk out	Trunk out	G	J
8/10/15	10:01	Thato	Trunk to arm prolonged	Prolonged contact	T	
8/10/15	10:05	Sally	Trunk to foot	Trunk to human	T	
8/10/15	10:05	Sally	Trunk to personal item	Trunk to object	T	
8/10/15	10:05	Sally	Trunk to leg	Trunk to human	G	F
8/10/15	12:09	Mashudu	Trunk out	Trunk out	T	
8/10/15	12:12	Sally	Trunk to bullhook	Trunk to object	G	H
8/10/15	12:13	Thato	Trunk to hand prolonged	Prolonged contact	T	
8/10/15	12:14	Mashudu	Trunk out	Trunk out	T	
8/10/15	12:14	Sally	Trunk out	Trunk out	G	H

8/10/15	12:26	Thandi	Trunk out	Trunk out	G	H
8/10/15	12:45	Thandi	Trunk to body	Trunk to human	G	A
8/10/15	13:29	Shungu	Approach	Seeking out	G	E
8/10/15	13:29	Shungu	Trunk out	Trunk out	G	E
8/10/15	14:55	Nandi	Trunk out	Trunk out	G	F
8/10/15	14:55	Sally	Trunk out	Trunk out	G	F
8/10/15	14:55	Sally	Trunk to hand	Trunk to human	G	F
8/10/15	14:57	Thandi	Trunk out	Trunk out	G	H
8/10/15	14:57	Sally	Trunk to leg	Trunk to human	T	
8/10/15	14:58	Nandi	Trunk out	Trunk out	G	J
8/10/15	15:24	Thato	Trunk out	Trunk out	T	
8/10/15	15:57	Thandi	Trunk to leg	Trunk to human	G	J
8/10/15	16:12	Mashudu	Approach	Seeking out	G	F
8/10/15	16:15	Mashudu	Approach	Seeking out	G	F
8/10/15	16:15	Mashudu	Trunk out	Trunk out	G	F
8/10/15	16:28	Mashudu	Approach	Seeking out	V	
8/10/15	16:28	Mashudu	Trunk to hand	Trunk to human	V	
8/10/15	16:28	Mashudu	Trunk to hand	Trunk to human	V	NR
8/10/15	16:28	Mashudu	Trunk to leg	Trunk to human	V	
8/10/15	16:28	Mashudu	Trunk to arm	Trunk to human	V	
8/10/15	16:40	Thato	Trunk out	Trunk out	G	B
8/10/15	16:41	Shungu	Approach	Seeking out	G	D
8/10/15	16:41	Shungu	Trunk to personal item	Trunk to object	V	
8/11/15	8:48	Sally	Trunk out	Trunk out	T	
8/11/15	8:48	Sally	Trunk to foot	Trunk to human	T	
8/11/15	8:50	Keisha	Trunk out	Trunk out	V	
8/11/15	8:53	Sally	Trunk to personal item	Trunk to object	T	
8/11/15	8:53	Keisha	Trunk to hand	Trunk to human	G	A
8/11/15	8:58	Shungu	Trunk out	Trunk out	T	

8/11/15	9:02	Thandi	Trunk to bullhook	Trunk to object	G	I
8/11/15	9:03	Keisha	Face	Other	G	I
8/11/15	10:14	Thandi	Approach	Seeking out	G	H
8/11/15	10:14	Thandi	Trunk to body	Trunk to human	G	H
8/11/15	10:16	Thandi	Trunk to personal item	Trunk to object	G	H
8/11/15	10:18	Thandi	Trunk out	Trunk out	G	H
8/11/15	10:18	Nandi	Trunk to body	Trunk to human	G	H
8/11/15	10:18	Thandi	Trunk to personal item	Trunk to object	G	H
8/11/15	10:18	Thandi	Trunk to hand	Trunk to human	G	H
8/11/15	12:31	Thandi	Trunk to body	Trunk to human	G	H
8/11/15	12:31	Thandi	Trunk out	Trunk out	G	H
8/11/15	12:32	Sally	Trunk out	Trunk out	G	I
8/11/15	12:34	Sally	Trunk to leg	Trunk to human	T	
8/11/15	12:36	Thandi	Trunk to body	Trunk to human	G	H
8/11/15	12:36	Nandi	Trunk out	Trunk out	T	
8/11/15	12:36	Thandi	Approach	Seeking out	G	H
8/11/15	12:38	Sally	Trunk out	Trunk out	G	E
8/11/15	12:38	Thandi	Head-to	Other	G	H
8/11/15	12:40	Keisha	Trunk to hand	Trunk to human	G	I
8/11/15	12:44	Thandi	Trunk hit	Other	T	
8/11/15	12:46	Keisha	Head-to	Other	G	I
8/11/15	12:55	Shungu	Trunk to body	Trunk to human	V	
8/11/15	14:30	Shungu	Approach	Seeking out	V	
8/11/15	14:30	Shungu	Trunk out	Trunk out	V	
8/11/15	14:51	Keisha	Trunk to personal item	Trunk to object	T	
8/11/15	15:15	Mashudu	Trunk to personal item	Trunk to object	V	
8/11/15	15:50	Thandi	Approach	Seeking out	G	E
8/11/15	16:42	Thandi	Approach	Seeking out	G	H
8/11/15	16:42	Thandi	Trunk out	Trunk out	G	H

8/12/15	9:51	Mashudu	Trunk out	Trunk out	V	
8/12/15	9:57	Thato	Trunk to bullhook	Trunk to object	G	I
8/12/15	15:58	Keisha	Approach	Seeking out	G	J
8/12/15	16:14	Shungu	Trunk out	Trunk out	G	J
8/12/15	16:33	Thandi	Trunk out	Trunk out	G	E
8/12/15	16:33	Thandi	Trunk to foot	Trunk to human	G	E
8/12/15	16:34	Nandi	Trunk out	Trunk out	G	E
8/12/15	16:34	Nandi	Trunk to leg	Trunk to human	G	E
8/12/15	16:38	Mashudu	Trunk out	Trunk out	G	J
8/14/15	10:24	Mashudu	Approach	Seeking out	G	F
8/14/15	10:24	Mashudu	Trunk out	Trunk out	G	F
8/14/15	10:26	Shungu	Trunk out	Trunk out	T	
8/14/15	10:26	Shungu	Trunk out	Trunk out	T	
8/14/15	10:29	Mashudu	Trunk out	Trunk out	G	F
8/15/15	9:52	Keisha	Trunk out	Trunk out	G	F
8/15/15	9:52	Keisha	Trunk to leg	Trunk to human	T	
8/15/15	10:31	Mashudu	Trunk to hand	Trunk to human	G	Y
8/15/15	14:26	Mashudu	Kick	Other	V	
8/15/15	14:59	Sally	Trunk out	Trunk out	G	F
8/15/15	14:59	Mashudu	Trunk out	Trunk out	G	F
8/15/15	15:30	Shungu	Trunk to hand	Trunk to human	V	
8/15/15	15:30	Shungu	Trunk out	Trunk out	V	
8/15/15	15:32	Shungu	Trunk to bullhook	Trunk to object	G	F
8/15/15	15:32	Keisha	Trunk to body	Trunk to human	T	
8/15/15	15:34	Shungu	Approach	Seeking out	G	E
8/15/15	15:34	Shungu	Trunk out	Trunk out	G	E
8/16/15	10:18	Shungu	Approach	Seeking out	G	L
8/16/15	10:18	Shungu	Trunk out	Trunk out	G	L
8/16/15	10:18	Thato	Approach	Seeking out	G	L

8/16/15	10:21	Mashudu	Approach	Seeking out	G	L
8/16/15	10:21	Mashudu	Trunk to personal item	Trunk to object	V	
8/16/15	10:21	Mashudu	Other	Other	V	
8/16/15	10:57	Nandi	Trunk out	Trunk out	G	E
8/16/15	11:00	Thato	Trunk to bullhook	Trunk to object	G	E
8/16/15	11:04	Thato	Push	Other	T	
8/16/15	11:07	Thandi	Trunk out	Trunk out	G	A
8/16/15	11:07	Thandi	Trunk out	Trunk out	G	H
8/16/15	11:09	Nandi	Trunk out	Trunk out	G	A
8/16/15	11:10	Thandi	Trunk out	Trunk out	G	Y
8/16/15	11:10	Sally	Trunk to foot	Trunk to human	G	Y
8/16/15	11:10	Mashudu	Trunk to leg	Trunk to human	G	F
8/16/15	11:17	Thandi	Trunk out	Trunk out	G	F
8/16/15	11:18	Sally	Trunk to foot	Trunk to human	T	
8/16/15	11:19	Nandi	Trunk out	Trunk out	G	Y
8/16/15	11:19	Nandi	Trunk to foot	Trunk to human	G	Y
8/16/15	11:19	Nandi	Trunk out	Trunk out	G	F
8/16/15	11:30	Keisha	Trunk out	Trunk out	T	
8/16/15	14:05	Shungu	Trunk to hand	Trunk to human	T	
8/16/15	14:28	Sally	Trunk to hand	Trunk to human	G	I
8/16/15	14:32	Mashudu	Trunk to hand	Trunk to human	G	F
8/16/15	14:35	Mashudu	Trunk out	Trunk out	V	
8/16/15	14:35	Mashudu	Trunk to hand	Trunk to human	V	
8/16/15	14:57	Shungu	Trunk out	Trunk out	G	E
8/16/15	14:58	Shungu	Trunk out	Trunk out	T	
8/16/15	14:58	Shungu	Trunk out	Trunk out	G	H
8/17/15	10:31	Sally	Trunk out	Trunk out	G	E
8/17/15	10:31	Keisha	Trunk to leg	Trunk to human	G	Y
8/17/15	10:33	Sally	Trunk to bullhook	Trunk to object	G	E

8/17/15	12:26	Shungu	Trunk to arm	Trunk to human	V	
8/17/15	15:14	Shungu	Trunk out	Trunk out	G	Y
8/17/15	15:26	Sally	Trunk to hand	Trunk to human	G	E
8/17/15	15:41	Sally	Trunk to leg	Trunk to human	T	
8/17/15	15:41	Sally	Trunk to hand	Trunk to human	T	
8/17/15	15:42	Sally	Trunk out	Trunk out	G	E
8/17/15	15:42	Sally	Trunk to hand	Trunk to human	G	E
8/17/15	15:47	Sally	Trunk to bullhook	Trunk to object	G	E
8/17/15	15:47	Sally	Trunk to hand	Trunk to human	G	E
8/17/15	15:49	Sally	Trunk to bullhook	Trunk to object	G	E
8/17/15	15:54	Shungu	Trunk out	Trunk out	G	H
8/17/15	15:57	Nandi	Trunk to body	Trunk to human	G	H
8/17/15	15:58	Nandi	Trunk out	Trunk out	G	F
8/17/15	16:00	Mashudu	Trunk to hand	Trunk to human	V	
8/17/15	16:18	Thandi	Approach	Seeking out	G	H
8/17/15	16:18	Thandi	Trunk to body	Trunk to human	G	H
8/18/15	9:22	Nandi	Trunk out	Trunk out	G	F
8/18/15	9:29	Nandi	Approach	Seeking out	G	F
8/18/15	9:29	Nandi	Trunk out	Trunk out	G	F
8/18/15	9:30	Nandi	Trunk to leg	Trunk to human	G	F
8/18/15	9:41	Thato	Trunk out	Trunk out	G	J
8/18/15	9:41	Thato	Trunk to body	Trunk to human	G	J
8/18/15	9:41	Thato	Trunk to personal item	Trunk to object	G	J
8/18/15	9:42	Thandi	Trunk out	Trunk out	G	F
8/18/15	9:44	Shungu	Trunk to body	Trunk to human	G	E
8/18/15	9:44	Thato	Approach	Seeking out	V	
8/18/15	9:44	Thato	Trunk to hand prolonged	Prolonged contact	V	
8/18/15	9:44	Thato	Trunk to body prolonged	Prolonged contact	G	L
8/18/15	9:47	Thato	Trunk to arm prolonged	Prolonged contact	G	L

8/18/15	9:47	Sally	Trunk to leg	Trunk to human	G	J
8/18/15	9:47	Thandi	Trunk to leg	Trunk to human	G	J
8/18/15	9:46	Thandi	Trunk out	Trunk out	G	J
8/18/15	9:47	Nandi	Trunk out	Trunk out	G	F
8/18/15	9:47	Thato	Trunk out	Trunk out	G	E
8/18/15	9:47	Mashudu	Approach	Seeking out	G	I
8/18/15	9:47	Mashudu	Head-lean	Prolonged contact	G	I
8/18/15	9:48	Thandi	Trunk to bullhook	Trunk to object	G	E
8/18/15	9:49	Thandi	Trunk out	Trunk out	G	L
8/18/15	9:49	Thandi	Trunk out	Trunk out	G	E
8/18/15	9:50	Mashudu	Trunk to personal item	Trunk to object	V	
8/18/15	9:50	Shungu	Trunk out	Trunk out	V	
8/18/15	9:51	Shungu	Trunk to hand	Trunk to human	V	
8/18/15	12:19	Shungu	Approach	Seeking out	G	E
8/18/15	12:53	Keisha	Approach	Seeking out	G	I
8/18/15	14:18	Nandi	Approach	Seeking out	G	A
8/18/15	14:28	Mashudu	Approach	Seeking out	G	E
8/18/15	15:06	Thato	Push	Other	G	I
8/18/15	15:36	Shungu	Trunk out	Trunk out	V	
8/19/15	8:41	Thato	Trunk out	Trunk out	T	
8/19/15	9:38	Mashudu	Trunk out	Trunk out	G	F
8/19/15	9:38	Mashudu	Trunk out	Trunk out	G	F
8/19/15	9:41	Shungu	Approach	Seeking out	G	F
8/19/15	9:41	Shungu	Trunk to hand	Trunk to human	G	F
8/19/15	9:44	Shungu	Trunk to hand	Trunk to human	G	F
8/19/15	9:44	Nandi	Approach	Seeking out	G	F
8/19/15	9:44	Nandi	Trunk to body	Trunk to human	G	F
8/19/15	9:52	Keisha	Trunk out	Trunk out	T	
8/19/15	11:56	Thandi	Approach	Seeking out	G	H

8/19/15	11:56	Thandi	Hug	Prolonged contact	G	H
8/19/15	12:33	Nandi	Trunk to leg	Trunk to human	G	L
8/19/15	12:34	Shungu	Approach	Seeking out	G	H
8/19/15	12:34	Shungu	Trunk to leg	Trunk to human	G	H
8/19/15	12:34	Shungu	Trunk to hand	Trunk to human	V	
8/19/15	12:34	Keisha	Trunk to hand	Trunk to human	G	H
8/19/15	12:34	Shungu	Trunk to leg	Trunk to human	G	
8/19/15	12:51	Nandi	Trunk to hand	Trunk to human	G	H
8/19/15	12:58	Keisha	Trunk to body	Trunk to human	G	F
8/19/15	12:59	Keisha	Approach	Seeking out	G	H
8/19/15	13:00	Thandi	Trunk to leg	Trunk to human	G	F
8/19/15	13:00	Thandi	Trunk to bullhook	Trunk to object	G	F
8/19/15	13:02	Nandi	Trunk to personal item	Trunk to object	V	
8/19/15	13:50	Mashudu	Trunk out	Trunk out	T	
8/20/15	10:57	Keisha	Face	Other	G	H
8/20/15	10:57	Keisha	Trunk to body	Trunk to human	G	H
8/20/15	10:57	Keisha	Trunk to personal item	Trunk to object	V	
8/21/15	9:06	Shungu	Trunk out	Trunk out	T	
8/21/15	9:50	Nandi	Trunk to leg	Trunk to human	G	E
8/21/15	9:50	Nandi	Trunk to body	Trunk to human	G	E
8/21/15	9:50	Mashudu	Trunk out	Trunk out	G	I
8/21/15	9:53	Keisha	Trunk to body	Trunk to human	V	
8/21/15	9:53	Keisha	Trunk to body	Trunk to human	V	
8/21/15	9:53	Keisha	Trunk to body	Trunk to human	V	
8/21/15	9:54	Keisha	Approach	Seeking out	G	I
8/21/15	9:54	Keisha	Trunk to body	Trunk to human	G	I
8/21/15	9:54	Keisha	Trunk to leg	Trunk to human	G	I
8/21/15	9:54	Keisha	Trunk out	Trunk out	V	
8/21/15	9:54	Keisha	Trunk to hand	Trunk to human	V	

8/21/15	9:56	Keisha	Head-lean	Prolonged contact	G	I
8/21/15	10:27	Shungu	Trunk to arm	Trunk to human	V	
8/21/15	10:30	Sally	Trunk out	Trunk out	T	
8/21/15	12:28	Thandi	Approach	Seeking out	G	H
8/21/15	12:28	Thandi	Trunk to hand	Trunk to human	G	H
8/21/15	12:30	Mashudu	Approach	Seeking out	G	H
8/21/15	12:30	Mashudu	Trunk out	Trunk out	G	H
8/21/15	12:54	Keisha	Head-to	Other	G	Y
8/21/15	13:20	Thato	Approach	Seeking out	G	I
8/21/15	13:20	Thato	Trunk to body	Trunk to human	G	I
8/21/15	14:50	Mashudu	Approach	Seeking out	G	J
8/21/15	14:58	Shungu	Trunk out	Trunk out	G	Y
8/21/15	15:24	Thandi	Trunk to hand	Trunk to human	G	I
8/21/15	15:54	Shungu	Trunk out	Trunk out	G	E
8/21/15	15:57	Nandi	Trunk to body	Trunk to human	V	
8/21/15	15:57	Nandi	Face	Other	G	I
8/21/15	16:19	Shungu	Trunk to arm	Trunk to human	G	H
8/21/15	16:20	Keisha	Approach	Seeking out	G	I
8/21/15	16:20	Keisha	Trunk out	Trunk out	G	I
8/22/15	14:55	Nandi	Approach	Seeking out	G	F
8/22/15	15:17	Shungu	Trunk to hand	Trunk to human	G	I
8/22/15	15:17	Shungu	Trunk to leg	Trunk to human	V	
8/22/15	15:17	Shungu	Trunk to bullhook	Trunk to object	G	I
8/22/15	15:29	Keisha	Ear flap	Other	T	
8/22/15	15:36	Keisha	Trunk out	Trunk out	G	I
8/22/15	15:57	Keisha	Trunk out	Trunk out	G	C
8/22/15	15:58	Mashudu	Trunk out	Trunk out	G	I
8/22/15	16:18	Nandi	Trunk out	Trunk out	G	I
8/23/15	11:29	Sally	Trunk to leg	Trunk to human	T	

8/23/15	11:31	Keisha	Trunk to personal item	Trunk to object	T	
8/23/15	11:32	Thato	Trunk out	Trunk out	G	H
8/23/15	12:03	Sally	Head-to	Other	G	J
8/23/15	12:05	Shungu	Trunk out	Trunk out	T	
8/23/15	12:32	Shungu	Trunk out	Trunk out	G	F
8/23/15	12:57	Sally	Trunk to arm	Trunk to human	V	
8/23/15	12:57	Sally	Trunk to leg	Trunk to human	V	
8/23/15	12:59	Sally	Head-to	Other	G	H
8/23/15	13:08	Mashudu	Trunk out	Trunk out	G	F
8/23/15	13:22	Shungu	Trunk out	Trunk out	G	H
8/23/15	13:38	Shungu	Trunk to hand	Trunk to human	T	
8/23/15	13:50	Mashudu	Trunk to leg	Trunk to human	V	
8/23/15	13:50	Mashudu	Trunk to hand	Trunk to human	V	
8/23/15	13:50	Mashudu	Trunk to personal item	Trunk to object	V	
8/23/15	13:50	Mashudu	Trunk to hand	Trunk to human	V	
8/23/15	13:50	Mashudu	Trunk to leg	Trunk to human	G	I
8/23/15	15:48	Thandi	Trunk out	Trunk out	G	Y
8/23/15	15:52	Keisha	Trunk to leg	Trunk to human	G	Y
8/23/15	16:02	Thandi	Approach	Seeking out	G	H
8/23/15	16:02	Thandi	Trunk to body	Trunk to human	G	H
8/23/15	16:02	Thandi	Trunk out	Trunk out	G	F
8/23/15	16:02	Nandi	Trunk out	Trunk out	T	
8/23/15	16:05	Shungu	Trunk to head	Trunk to human	T	
8/23/15	16:05	Shungu	Trunk to body	Trunk to human	T	
8/23/15	16:06	Keisha	Trunk to body	Trunk to human	G	F
8/23/15	16:06	Sally	Trunk to body	Trunk to human	G	F
8/23/15	16:07	Sally	Trunk to leg	Trunk to human	G	F
8/23/15	16:07	Sally	Trunk to body	Trunk to human	G	F
8/23/15	16:07	Sally	Trunk to hand	Trunk to human	T	

8/23/15	16:08	Thandi	Trunk to body	Trunk to human	G	H
8/23/15	16:08	Nandi	Trunk out	Trunk out	G	H
8/23/15	16:09	Nandi	Approach	Seeking out	G	I
8/23/15	16:09	Nandi	Trunk to foot	Trunk to human	G	I
8/23/15	16:10	Shungu	Trunk to body	Trunk to human	G	I
8/23/15	16:11	Keisha	Head-to	Other	G	C
8/23/15	16:12	Keisha	Trunk out	Trunk out	T	
8/23/15	16:12	Keisha	Trunk to hand	Trunk to human	V	
8/24/15	8:49	Sally	Trunk to hand	Trunk to human	G	E
8/24/15	8:51	Mashudu	Trunk to foot	Trunk to human	V	
8/24/15	9:14	Thato	Face	Other	V	
8/24/15	14:16	Thato	Trunk out	Trunk out	G	C
8/24/15	14:55	Sally	Trunk to leg	Trunk to human	G	F
8/24/15	15:30	Nandi	Trunk out	Trunk out	G	E
8/25/15	13:29	Shungu	Trunk out	Trunk out	T	
8/25/15	14:10	Keisha	Approach	Seeking out	G	E
8/25/15	14:27	Shungu	Approach	Seeking out	G	E
8/25/15	14:27	Shungu	Trunk out	Trunk out	G	E
8/26/25	9:55	Nandi	Approach	Seeking out	G	H
8/26/15	10:05	Mashudu	Trunk out	Trunk out	G	E
8/26/05	10:05	Mashudu	Trunk out	Trunk out	G	F
8/26/95	10:06	Mashudu	Trunk out	Trunk out	G	E
8/26/85	10:23	Thandi	Trunk out	Trunk out	G	H
8/26/75	10:25	Shungu	Approach	Seeking out	G	E
8/26/65	10:25	Shungu	Trunk out	Trunk out	G	E
8/26/55	10:27	Shungu	Approach	Seeking out	G	L
8/26/45	10:27	Shungu	Trunk out	Trunk out	G	L
8/26/35	10:48	Thandi	Trunk to body	Trunk to human	G	E
8/26/25	10:48	Nandi	Trunk to body	Trunk to human	G	E

8/26/15	10:55	Shungu	Trunk out	Trunk out	G	H
8/26/05	10:57	Keisha	Trunk to hand	Trunk to human	G	F
8/26/15	10:59	Nandi	Trunk to hand	Trunk to human	G	H
8/26/25	10:59	Mashudu	Trunk to hand	Trunk to human	T	
8/26/15	11:23	Thandi	Approach	Seeking out	G	H
8/26/25	11:23	Thandi	Trunk to leg	Trunk to human	G	H
8/26/15	11:24	Sally	Trunk out	Trunk out	G	I
8/26/25	11:54	Sally	Trunk to hand	Trunk to human	G	I
8/26/15	11:55	Nandi	Trunk out	Trunk out	G	H
8/26/25	12:08	Thato	Trunk to hand	Trunk to human	T	
8/26/15	12:17	Shungu	Trunk out	Trunk out	T	
8/26/25	12:17	Shungu	Trunk out	Trunk out	G	I
8/26/15	12:19	Keisha	Trunk to hand	Trunk to human	G	I
8/26/25	12:24	Mashudu	Trunk out	Trunk out	G	I
8/26/15	12:25	Mashudu	Trunk out	Trunk out	G	I
8/26/25	12:26	Mashudu	Approach	Seeking out	G	Y
8/26/15	12:26	Mashudu	Trunk out	Trunk out	G	Y
8/26/15	12:27	Shungu	Trunk to hand	Trunk to human	V	
8/26/25	12:27	Shungu	Trunk to hand	Trunk to human	V	
8/26/15	12:32	Nandi	Trunk out	Trunk out	G	I
8/26/25	12:45	Nandi	Trunk to leg	Trunk to human	G	H
8/26/15	14:57	Keisha	Trunk to hand	Trunk to human	G	F
8/26/25	15:25	Thato	Trunk to hand	Trunk to human	T	
8/26/15	15:49	Mashudu	Approach	Seeking out	G	M
8/27/15	8:45	Sally	Trunk to body	Trunk to human	V	
8/27/15	9:04	Keisha	Trunk out	Trunk out	T	
8/27/15	13:24	Shungu	Trunk to hand prolonged	Prolonged contact	V	
8/27/15	13:48	Shungu	Approach	Seeking out	G	F
8/27/15	14:08	Mashudu	Trunk out	Trunk out	G	F

8/27/15	14:17	Shungu	Trunk to hand prolonged	Prolonged contact	V	
8/27/15	14:17	Shungu	Trunk to foot	Trunk to human	G	I
8/27/15	14:17	Shungu	Trunk to hand prolonged	Prolonged contact	G	I
8/27/15	14:17	Shungu	Trunk out	Trunk out	V	
8/27/15	14:39	Shungu	Approach	Seeking out	V	
8/27/15	14:39	Shungu	Trunk out	Trunk out	V	
8/27/15	14:54	Thandi	Trunk to body	Trunk to human	G	H
8/27/15	14:55	Shungu	Trunk out	Trunk out	G	E
8/27/15	14:56	Keisha	Trunk out	Trunk out	G	H
8/28/15	11:26	Mashudu	Trunk out	Trunk out	G	F
8/28/15	11:30	Keisha	Trunk out	Trunk out	T	
8/28/15	11:30	Keisha	Trunk out	Trunk out	T	
8/28/15	11:30	Keisha	Trunk out	Trunk out	G	B
8/28/15	11:32	Shungu	Trunk out	Trunk out	G	B
8/28/15	11:33	Shungu	Trunk to personal item	Trunk to object	T	
8/28/15	11:33	Shungu	Trunk out	Trunk out	T	
8/28/15	11:33	Shungu	Trunk to body	Trunk to human	G	B
8/28/15	11:40	Shungu	Trunk to body	Trunk to human	G	B
8/28/15	11:40	Shungu	Trunk to body	Trunk to human	G	B
8/28/15	11:54	Nandi	Approach	Seeking out	G	F
8/28/15	11:54	Nandi	Trunk to bullhook	Trunk to object	G	F
8/28/15	11:54	Nandi	Trunk to hand	Trunk to human	G	I
8/28/15	11:55	Shungu	Trunk to bullhook	Trunk to object	G	J
8/28/15	11:58	Thato	Approach	Seeking out	G	F
8/28/15	12:00	Thato	Trunk out	Trunk out	G	J
8/28/15	12:21	Sally	Trunk to foot	Trunk to human	T	
8/28/15	12:55	Keisha	Trunk to hand	Trunk to human	G	I
8/28/15	12:55	Keisha	Trunk to body	Trunk to human	G	I
8/28/15	12:55	Keisha	Trunk to hand	Trunk to human	G	I

8/28/15	13:01	Keisha	Trunk to hand	Trunk to human	V	
8/28/15	13:01	Keisha	Trunk to body	Trunk to human	G	I
8/28/15	13:01	Keisha	Trunk to hand	Trunk to human	G	I
8/28/15	13:01	Keisha	Trunk to personal item	Trunk to object	V	
8/28/15	16:36	Thato	Trunk to hand	Trunk to human	G	L
8/29/15	9:26	Nandi	Trunk out	Trunk out	G	I
8/29/15	9:26	Shungu	Approach	Seeking out	G	D
8/29/15	9:27	Shungu	Turn body	Other	G	I
8/29/15	9:27	Thandi	Trunk out	Trunk out	G	I
8/29/15	9:28	Thandi	Trunk to body	Trunk to human	G	I
8/29/15	9:28	Thandi	Trunk out	Trunk out	G	I
8/29/15	9:37	Shungu	Follow	Seeking out	G	E
8/29/15	9:54	Sally	Trunk to hand	Trunk to human	T	
8/29/15	10:56	Thandi	Approach	Seeking out	G	K
8/29/15	11:20	Shungu	Trunk to body	Trunk to human	G	H
8/29/15	11:25	Mashudu	Trunk to hand	Trunk to human	G	D
8/29/15	11:28	Mashudu	Trunk to body	Trunk to human	V	
8/29/15	11:28	Mashudu	Trunk to hand	Trunk to human	V	
8/29/15	11:31	Mashudu	Trunk to hand	Trunk to human	V	
8/29/15	11:45	Thandi	Trunk to hand	Trunk to human	G	H
8/29/15	12:59	Keisha	Trunk to foot	Trunk to human	G	D
8/29/15	13:58	Keisha	Trunk out	Trunk out	T	
8/29/15	14:03	Mashudu	Trunk to hand	Trunk to human	V	
8/29/15	14:03	Mashudu	Follow	Seeking out	G	I
8/29/15	14:03	Mashudu	Trunk to hand	Trunk to human	V	
8/29/15	14:11	Sally	Trunk to body	Trunk to human	G	E
8/29/15	14:17	Thandi	Trunk to body	Trunk to human	T	
8/29/15	14:29	Shungu	Trunk to hand	Trunk to human	G	E
8/29/15	14:29	Shungu	Trunk to hand	Trunk to human	T	

8/29/15	15:20	Shungu	Trunk to hand	Trunk to human	T	
8/29/15	15:45	Thato	Approach	Seeking out	G	L
8/29/15	15:45	Thato	Trunk out	Trunk out	G	L
8/29/15	15:46	Nandi	Trunk to foot	Trunk to human	G	J
8/29/15	15:47	Thato	Trunk to body	Trunk to human	G	H
8/29/15	15:56	Keisha	Trunk to personal item	Trunk to object	T	
8/29/15	15:56	Sally	Trunk to foot	Trunk to human	T	
8/29/15	16:20	Sally	Approach	Seeking out	G	D
8/30/15	11:55	Keisha	Trunk to body	Trunk to human	T	
8/30/15	12:31	Shungu	Trunk to hand	Trunk to human	V	
8/30/15	12:45	Thandi	Trunk to hand	Trunk to human	V	
8/30/15	12:45	Thandi	Trunk to body	Trunk to human	G	H
8/30/15	12:45	Thandi	Trunk out	Trunk out	V	
8/31/15	9:26	Keisha	Trunk out	Trunk out	T	
8/31/15	9:35	Shungu	Trunk to hand	Trunk to human	V	
8/31/15	9:57	Nandi	Trunk out	Trunk out	G	E
8/31/15	10:53	Shungu	Trunk out	Trunk out	V	
8/31/15	10:53	Sally	Trunk out	Trunk out	T	
8/31/15	10:56	Nandi	Approach	Seeking out	G	E
8/31/15	10:56	Nandi	Trunk out	Trunk out	G	E
8/31/15	11:04	Thandi	Approach	Seeking out	G	E
9/2/15	12:39	Mashudu	Trunk out	Trunk out	G	I
9/2/15	12:39	Mashudu	Trunk out	Trunk out	G	I
9/2/15	13:08	Keisha	Trunk to hand	Trunk to human	G	Y
9/2/15	13:08	Keisha	Trunk to body	Trunk to human	G	Y
9/2/15	13:08	Keisha	Trunk out	Trunk out	G	Y
9/2/15	13:08	Keisha	Trunk out	Trunk out	G	Y
9/2/15	14:02	Shungu	Trunk to hand	Trunk to human	V	
9/2/15	14:02	Shungu	Trunk to hand	Trunk to human	V	

9/2/15	14:02	Shungu	Trunk to hand	Trunk to human	V	
9/2/15	14:02	Shungu	Trunk to leg	Trunk to human	V	
9/2/15	14:04	Keisha	Trunk to body	Trunk to human	T	
9/2/15	14:16	Shungu	Trunk to hand	Trunk to human	V	
9/3/15	9:27	Sally	Trunk out	Trunk out	G	H
9/3/15	9:30	Sally	Head-to	Other	G	H
9/3/15	10:26	Shungu	Trunk to hand	Trunk to human	G	E
9/3/15	10:26	Shungu	Follow	Seeking out	G	E
9/3/15	10:26	Shungu	Trunk to leg	Trunk to human	G	E
9/3/15	10:28	Thandi	Trunk to bullhook	Trunk to object	G	J
9/3/15	10:41	Sally	Trunk to leg	Trunk to human	T	
9/3/15	10:41	Sally	Trunk to body	Trunk to human	T	
9/3/15	10:42	Sally	Trunk to personal item	Trunk to object	T	
9/4/15	10:27	Keisha	Trunk out	Trunk out	G	E
9/4/15	10:27	Keisha	Trunk out	Trunk out	G	A
9/4/15	11:24	Nandi	Approach	Seeking out	G	A
9/4/15	11:25	Sally	Trunk to foot	Trunk to human	G	Y
9/4/15	11:25	Sally	Trunk to leg	Trunk to human	G	Y
9/4/15	11:27	Sally	Trunk out	Trunk out	T	
9/4/15	13:23	Mashudu	Trunk out	Trunk out	T	
9/4/15	13:24	Shungu	Trunk to hand	Trunk to human	V	
9/4/15	13:24	Thato	Trunk out	Trunk out	G	M
9/4/15	14:27	Shungu	Trunk out	Trunk out	G	E
9/4/15	14:27	Shungu	Trunk to leg	Trunk to human	G	E
9/4/15	14:32	Thandi	Trunk out	Trunk out	G	E
9/4/15	14:37	Mashudu	Approach	Seeking out	V	
9/4/15	14:37	Mashudu	Trunk to body	Trunk to human	V	
9/4/15	15:00	Shungu	Trunk to hand	Trunk to human	V	
9/5/15	10:26	Shungu	Trunk out	Trunk out	G	M

9/5/15	10:26	Shungu	Trunk out	Trunk out	T	
9/5/15	10:28	Shungu	Trunk out	Trunk out	T	
9/5/15	10:33	Keisha	Approach	Seeking out	G	K
9/5/15	10:38	Nandi	Face	Other	G	D
9/5/15	10:38	Nandi	Trunk out	Trunk out	G	D
9/5/15	10:42	Thato	Trunk out	Trunk out	G	D
9/5/15	10:42	Thato	Trunk out	Trunk out	V	
9/5/15	10:42	Thato	Trunk to arm	Trunk to human	V	
9/5/15	11:05	Shungu	Trunk to hand	Trunk to human	G	D
9/5/15	11:07	Nandi	Trunk out	Trunk out	G	D
9/5/15	11:14	Nandi	Trunk to arm	Trunk to human	G	I
9/5/15	11:14	Nandi	Trunk to bullhook	Trunk to object	G	I
9/5/15	11:35	Thato	Trunk to hand	Trunk to human	G	I
9/5/15	11:35	Thato	Trunk to hand	Trunk to human	G	I
9/5/15	11:45	Shungu	Trunk out	Trunk out	G	A
9/5/15	13:26	Sally	Trunk to leg	Trunk to human	G	E
9/5/15	13:27	Sally	Trunk out	Trunk out	G	E
9/5/15	13:27	Sally	Trunk to bullhook	Trunk to object	G	E
9/5/15	13:29	Sally	Trunk to leg	Trunk to human	G	E
9/5/15	13:29	Sally	Trunk out	Trunk out	G	E
9/5/15	13:30	Keisha	Trunk out	Trunk out	T	
9/5/15	13:44	Thandi	Trunk to personal item	Trunk to object	V	
9/5/15	13:44	Thandi	Trunk to hand	Trunk to human	V	
9/5/15	14:27	Shungu	Trunk out	Trunk out	G	E
9/5/15	14:28	Shungu	Trunk out	Trunk out	G	E
9/5/15	14:29	Shungu	Trunk to hand	Trunk to human	V	
9/5/15	14:29	Shungu	Trunk to hand	Trunk to human	V	
9/5/15	14:29	Shungu	Trunk to body	Trunk to human	V	
9/5/15	14:29	Shungu	Trunk out	Trunk out	G	E

9/5/15	14:37	Thato	Trunk to personal item	Trunk to object	V	
9/5/15	14:37	Thato	Trunk to hand	Trunk to human	V	
9/5/15	14:46	Sally	Trunk to leg	Trunk to human	V	
9/5/15	15:06	Keisha	Trunk out	Trunk out	G	C
9/5/15	15:07	Keisha	Trunk to leg	Trunk to human	G	D
9/5/15	15:43	Nandi	Trunk to hand	Trunk to human	V	
